# Maternal cardiovascular health and offspring neurodevelopment within the first five years of life: a birth cohort study

**DOI:** 10.1007/s12519-025-00969-5

**Published:** 2025-09-19

**Authors:** Han Qiu, Chun-Yan Zhou, Shou-Xun Hu, Luan-Luan Li, Xi-Rui Wang, Jun Zhang, Ying Tian, Bin Wang, Xiao-Dan Yu

**Affiliations:** 1 Department of Developmental and Behavioral Pediatrics, Shanghai Children’s Medical Center, School of Medicine, Shanghai Jiao Tong University, 1678 Dongfang Road, Shanghai, 200127 China; 2 Translational Medicine Institute, Shanghai Children’s Medical Center, School of Medicine, Shanghai Jiao Tong University, Shanghai, 200127 China; 3 MOE-Shanghai Key Lab of Children’s Environmental Health, Xinhua Hospital, School of Medicine, Shanghai Jiao Tong University, Shanghai, 200092 China; 4 Department of Endocrinology and Metabolism, Shanghai Ninth People’s Hospital, School of Medicine, Shanghai Jiao Tong University, Shanghai, 200092 China

**Keywords:** Birth cohort, Gestation, Longitudinal study, Maternal cardiovascular health, Offspring neurodevelopment

## Abstract

**Background:**

The first five years of life are sensitive periods for neurodevelopment. Poor maternal metrics of cardiovascular health may influence offspring neurodevelopment. Previous studies focused only on one or two metrics, or different time window. This study is aimed to investigate the effects of combined cardiovascular health metric exposure during pregnancy on the neurodevelopment of offspring during crucial periods.

**Methods:**

A total of 1007 mother‒child pairs recruited from 2013 to 2016 from the Shanghai Birth Cohort were included. Five maternal cardiovascular health metrics at 28 weeks of gestation were collected. Offspring neurodevelopment at 2–3 years and 4–5 years was evaluated with the Bayley-III and Wechsler preschool and primary scale of intelligence, fourth edition (WPPSI-IV), respectively.

**Results:**

After adjusting for confounders, the scores for cognition and language at 2–3 years significantly increased by 1.63 [95% confidence interval (CI) 0.42–2.83, *P* = 0.008] and 0.84 (95% CI 0.005–1.67, *P* = 0.049) per one-point higher maternal cardiovascular health score, respectively. After false discovery rate adjustment, the associations were preserved in the cognitive domain. Similarly, each one-point higher maternal cardiovascular health score was associated with an increase of 0.92 (95% CI 0.16–1.68, *P* = 0.018) and 0.71 (95% CI 0.01–1.40, *P* = 0.047) in the visual space index and working memory index scores at 4–5 years, respectively, but with an false discovery rate-adjusted *P* > 0.05; in the sex-stratified analysis, the visual space index scores significantly increased (*β* = 1.47, 95% CI 0.38–2.56, *P* = 0.009), regardless of false discovery rate correction. In addition, each one-point higher maternal cardiovascular health score reduced the relative risk of suboptimal development in the visual space index domain by 0.83 (95% CI 0.70–0.99; *P* = 0.041) in female offspring despite the non-significant after false discovery rate adjustment.

**Conclusions:**

Our study provides novel evidence that maternal cardiovascular health during pregnancy is associated with offspring neurodevelopment within the first five years of life and that female offspring appear to derive greater benefit from higher maternal cardiovascular health scores. The potential role of maternal cardiovascular health in identifying risk of neurodevelopmental delay in clinical practice needs to be further explored.

**Graphical abstract:**

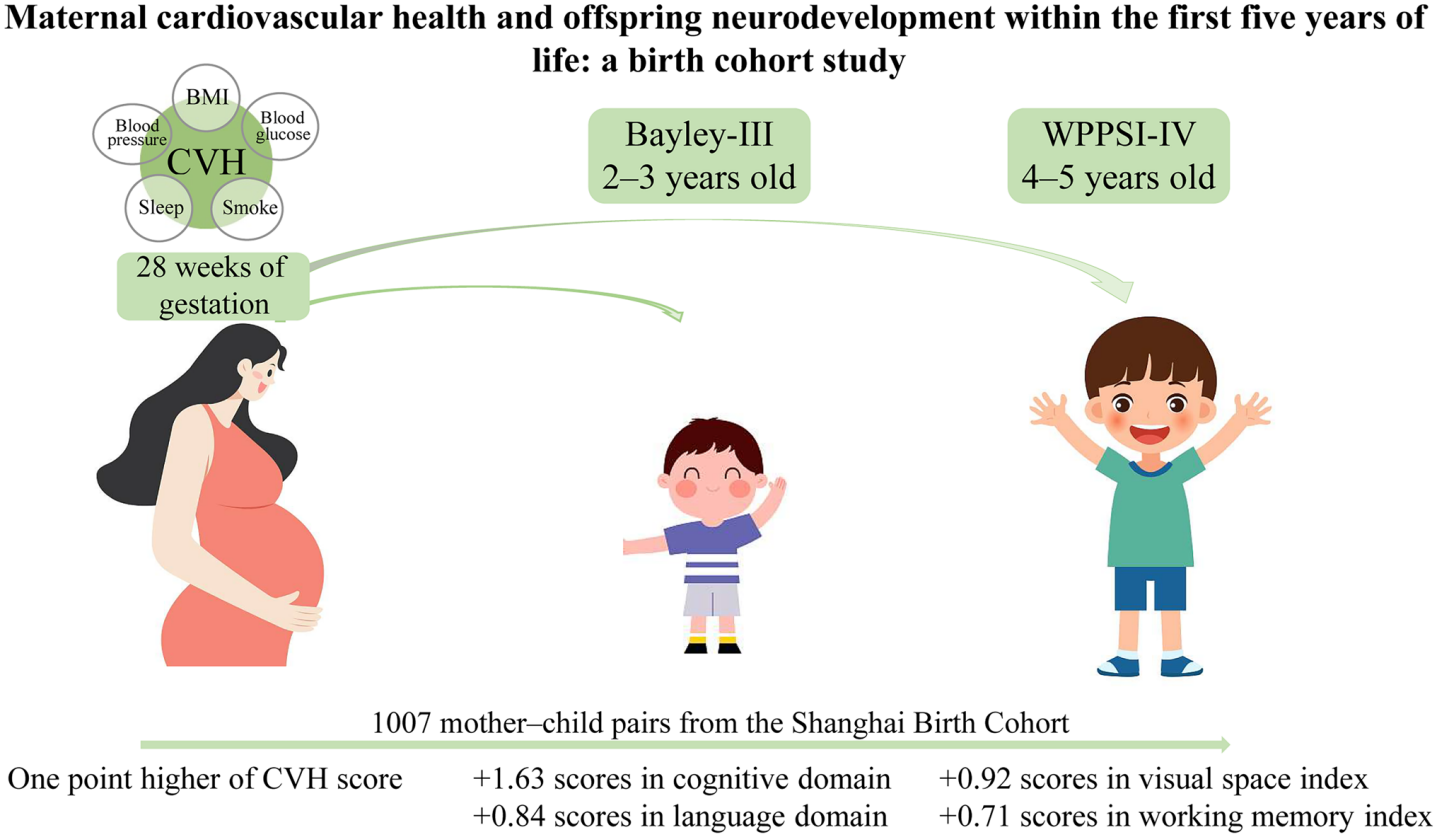

**Supplementary Information:**

The online version contains supplementary material available at 10.1007/s12519-025-00969-5.

## Introduction

Cardiovascular health (CVH), including body mass index (BMI), blood pressure, total cholesterol level, glucose level, smoking, diet, sleep, and physical activity [1–3], is defined by the American Heart Association to identify pregnant women at high risk for cardiovascular disease [4]. The developmental origins of health and disease emphasize that gestation is a key period for offspring brain development [5]. During gestation, maternal exposure to poor metrics of CVH in utero may lead to a higher-risk trajectory for brain development and increase the risk for the development of other diseases, including obesity [6], diabetes [7], and hypertensive disorders [8], in offspring. Recently, some studies have reported that single CVH metrics are associated with offspring neurodevelopment [9–15]. However, previous studies focused on only one or two CVH metrics of exposure during pregnancy. To date, only one study from Hefei City, China, has shown that poor combined maternal CVH metrics (BMI, blood pressure, total cholesterol, glucose level, and smoking) at 24–28 weeks gestation are associated with an increased risk of failure in the communication domain [16]. However, in this study, children were followed up to only one year of age. The first five years of life is a sensitive window for neurodevelopment during which the human brain rapidly develops, with about 90% of brain growth occurring during this period [17]. Hence, it is worth studying the impact of maternal gestational CVH metrics during pregnancy, particularly combined metrics, on offspring neurodevelopment during the critical period.

To fill this knowledge gap, the current study combined five metrics of CVH, including BMI, blood pressure, glucose level, sleep and smoking, and recruited mother‒child pairs from the Shanghai Birth Cohort (SBC) to elucidate the effects of combined maternal CVH during pregnancy on offspring neurodevelopment at 2–3 and 4–5 years of age.

## Methods

### Participants and study design

We obtained data from the SBC, which is a prospective cohort study established in Shanghai, China, from 2013 to 2016. The details were published previously [18]. Briefly, pregnant women who participated in the SBC visit hospitals during each trimester and at delivery. Sociodemographic characteristics, including maternal age, prepregnant BMI, education, mood (anxiety, depression, and pressure), etc., and infant sex and birth weight were collected via a questionnaire from all participants via face-to-face interviews at the second trimester visit or delivery. The neurodevelopment of the offspring at 2–3 and 4–5 years of age was evaluated with the Bayley scales of infant and toddler development, third edition (Bayley-III) and the Wechsler preschool and primary scale of intelligence, fourth edition (WPPSI-IV), respectively.

The inclusion criterion was singleton live births with pregnant women aged ≥ 18 years who delivered at ≥ 37 gestational weeks without receiving assisted reproductive technology. Pregnant women without BMI, blood pressure, glucose level, sleep and smoking information; newborns with major malformations; and children without completion of the Bayley-III or WPPSI-IV assessments were excluded. Eventually, 1033 pregnant women met the criteria, and 1007 children finished the neurodevelopment assessment at two time points. Altogether, 1007 mother‒child pairs were included in the final analysis (Supplementary Fig. 1).

This study was approved by the Medical Ethics Committee of Shanghai Xinhua Hospital affiliated with Shanghai Jiao Tong University School of Medicine (XHEC-C-2013-001-3). Written informed consent was obtained from mothers for themselves and their children.

### Maternal gestational cardiovascular health

BMI, blood pressure, blood glucose, sleep, and smoking data were acquired from pregnant women at 28 weeks gestation (24–32 weeks). Height, weight, and blood pressure were measured twice by trained personnel via calibrated instruments. BMI was calculated as weight in kilograms divided by height in square meters. Venous blood was drawn after fasting and one and two hours after a 75 g oral glucose load and was sent to the Clinical Laboratory Centre of Xin Hua Hospital, Shanghai Jiao Tong University School of Medicine, for analysis. Sleep status was measured with the Pittsburgh sleep quality index (PSQI), a self-rated questionnaire scored from 0 to 21. Smoking status data were collected via questionnaire.

Each CVH metric was classified as ideal, intermediate, or poor on the basis of pregnancy guidelines (Supplementary Table 1). According to the World Health Organization categories for nonpregnant adults and gestational weight gain [19, 20], BMI thresholds ≤ 28.4 kg/m^2^ were defined as ideal, 28.5–32.9 kg/m^2^ as intermediate, and ≥ 33 kg/m^2^ as poor. Blood pressure thresholds [systolic blood pressure (SBP) < 120 mmHg and diastolic blood pressure (DBP) < 80 mmHg (ideal), SBP 120–139 mmHg or DBP 80–89 mmHg (intermediate), and SBP ≥ 140 or DBP ≥ 90 (poor)] were identified via pregnancy guidelines [21]. For sleep status, a PSQI score ≤ 5 was defined as ideal, a score of 6–9 was defined as intermediate, and a score of ≥ 10 was defined as poor [22, 23]. For glucose level and smoking status, poor status was defined for each patient on the basis of guidelines, and all others were considered to have ideal status [24]. A poor glucose level was defined as gestational diabetes (International Association of Diabetes in Pregnancy Study Groups/World Health Organization criteria: fasting blood glucose (FBG) ≥ 5.1 mmol/L, or one-hour plasma glucose level in a 75 g oral glucose tolerance test (OGTT) ≥ 10.0 mmol/L, or two-hour plasma glucose level in a 75 g OGTT ≥ 8.5 mmol/L), and poor smoking status was defined as current smoking (at 24–32 weeks gestation) [24, 25]. Each of the five metrics was assigned two points for ideal, one for intermediate, and zero for poor levels, yielding a total CVH score ranging from 0 to 10 points. Total CVH scores were further categorized into four groups: ideal-only (all metrics ideal), intermediate-only (≥ 1 intermediate, no poor), one-poor, and multiple-poor (≥ 2 poor) [20].

### Neurodevelopment assessments

Neurodevelopment at 2–3 years of age was assessed with the Chinese version of the Bayley-III, which is a comprehensive assessment tool for infants and toddlers aged 1–42 months and includes five independent subscales, i.e., the cognition, language, motor, social–emotional, and adaptive behavior subscales [26]. Neurodevelopment at 4–5 years of age was evaluated with the WPPSI-IV, which is a widely used test to measure intellectual ability in children aged 2.6–7.3 years and consists of the verbal comprehension index (VCI), visual space index (VSI), fluid reasoning index (FRI), working memory index (WMI), processing speed index (PSI), and full-scale IQ (FSIQ) [27].

The Bayley-III and WPPSI-IV raw scores were converted into composite scores and scaled to a metric with a mean of 100 and a standard deviation (SD) of 15. Scores in the lowest quartile for all the Bayley-III subscales and the WPPSI-IV were defined as suboptimal development [28].

### Statistical analysis

The participants’ characteristics are presented as frequencies and proportions for categorical variables and means and SDs for continuous variables. Before linear regression analysis, normality assumption and linearity were measured with graphical methods, and the multicollinearity variance was examined with inflation factors that exceeded four, indicating problematic collinearity. A linear regression model was applied to examine the associations of demographic characteristics with offspring neurodevelopment. A multiple linear regression model was used to evaluate the associations of combined maternal CVH levels with Bayley-III and WPPSI-IV scores. The risk of suboptimal neurodevelopment exposure to maternal CVH was assessed with a multiple Poisson regression model. Considering the multiple comparisons, the Benjamini‒Hochberg procedure was used to control the false discovery rate (FDR) [29, 30]. Given that the effect of maternal CVH on neurodevelopment could differ by sex [31–33], a sex-stratified analysis was conducted. In addition, the relationships between maternal CVH status and offspring neurodevelopment at two time points were also analyzed. On the basis of previous studies [28] and univariable analysis, maternal age, prepregnant BMI, education, mood (anxiety, depression, and pressure), infants’ sex and birth weight were considered confounders in all adjusted models.

Missing values for covariates (< 5%) were imputed by multivariable imputation via chained equations with the R package “mice” (version 4.3.1). All the statistical analyses were performed in R version 4.3.1. A two-sided *P* value < 0.05 was considered statistically significant.

## Results

### Demographic characteristics and neurodevelopment scores

The general demographic characteristics of 1007 mother‒child pairs are shown in Table 1. The mean (SD) maternal age at delivery was 28.84 (3.67) years. A total of 90.8% of the mothers had an education level above a high school degree. The average (SD) birthweight was 3426.53 (419.36) g, and 53.1% of the newborns were male. The average (SD) maternal CVH score was 8.05 (1.27); 8.2% of mothers were in the ideal-only group (*n* = 83), and 5.9% were in the multiple-poor group (*n* = 59).

**Table 1:** Participant characteristics

Characteristics	Statistics, mean (SD) or number (%)
Number	1007
Maternal information	
BMI (kg/m^2^)	27.03 (3.19)
Ideal	693 (68.8%)
Intermediate	268 (26.6%)
Poor	46 (4.6%)
Blood glucose (mmol/L)	
FBG	4.41 (0.47)
1 h after a 75-g oral glucose load	7.63 (1.68)
2 h after a 75-g oral glucose load	6.51 (1.43)
Ideal	857 (85.1%)
Intermediate	/
Poor	150 (14.9%)
Blood pressure (mmHg)	
Systolic pressure	126.03 (10.30)
Diastolic pressure	76.21 (8.75)
Ideal	176 (17.5%)
Intermediate	689 (68.4%)
Poor	142 (14.1%)
PSQI total score	4.59 (2.46)
Ideal	700 (69.5%)
Intermediate	286 (28.4%)
Poor	21 (2.1%)
Smoke	
Ideal	1006 (99.9%)
Intermediate	/
Poor	1 (0.1%)
CVH score	8.05 (1.27)
Ideal-only	83 (8.2%)
Intermediate-only	601 (59.7%)
One-poor	264 (26.2%)
Multiple-poor	59 (5.9%)
Maternal age (y)	28.84 (3.67)
Pre-pregnant BMI (kg/m^2^)	22.29 (3.07)
Missing	2 (0.2%)
Maternal education	
Below high school degree	27 (2.7%)
High school degree	63 (6.3%)
College degree	262 (26.0%)
Bachelor’s degree or above	653 (64.8%)
Missing	2 (0.2%)
Anxiety score	30.43 (5.42)
Missing	17 (1.7%)
Depression score	10.18 (7.07)
Missing	28 (2.8%)
Pressure score	12.20 (5.10)
Missing	14 (1.4%)
Offspring information	
Gestational weight gain (g)	3426.53 (419.36)
Missing	1 (0.1%)
Infant sex	
Male	535 (53.1%)
Female	472 (46.9%)

/ not available, *SD* standard deviation, *BMI* body mass index, *FBG* fasting blood glucose, *PSQI* Pittsburgh sleep quality index, *CVH* cardiovascular health, *WPPSI* Wechsler preschool and primary scale of intelligence, *IQ* intelligence quotient

Mean (SD) cognition, language, motor, social–emotional, and adaptive behavior subscale scores assessed at 2–3 years of age were 113 (22.7), 95.6 (15.5), 107 (15.0), 105 (18.1) and 105 (19.1), respectively. The means (SDs) of the VCI, VSI, FRI, WMI, PSI, and FSIQ scores assessed at 4–5 years of age were 115 (13.6), 113 (13.6), 110 (12.1), 105 (12.3), 106 (11.4), and 114 (11.9), respectively (Supplementary Table 2). Since studies reported that the Bayley-III domains were related to the WPPPSI-IV domains [34], the correlation between each domain in the two scales was evaluated (Supplementary Fig. 2). Our results revealed that each domain of the Bayley-III was significantly correlated with respective domain of the WPPSI-IV (*P* < 0.001).

### Single maternal cardiovascular health metrics and offspring neurodevelopment

The associations of each metric of maternal gestational CVH with neurodevelopment at 2–3, and 4–5 years of age were examined (Supplementary Tables 3–8). The results showed that in both the unadjusted and adjusted models, cognitive and language development at 2–3 years of age were significantly associated with blood glucose, blood pressure and sleep; motor development at 2–3 years of age was significantly associated with blood pressure and sleep (Supplementary Tables 5 and 7). The results of the WPPIS-IV at 4–5 years of age showed that in the unadjusted model, VCI and VSI were significantly associated with blood glucose; VCI, PSI, and FSIQ were significantly associated with blood pressure; and FRI was significantly associated with sleep (Supplementary Table 6). In the adjusted model, PSI was significantly associated with blood pressure (Supplementary Table 8).

### Combined maternal cardiovascular health metrics and offspring neurodevelopment

#### Neurodevelopment at 2–3 years of age

**Fig. 1:**
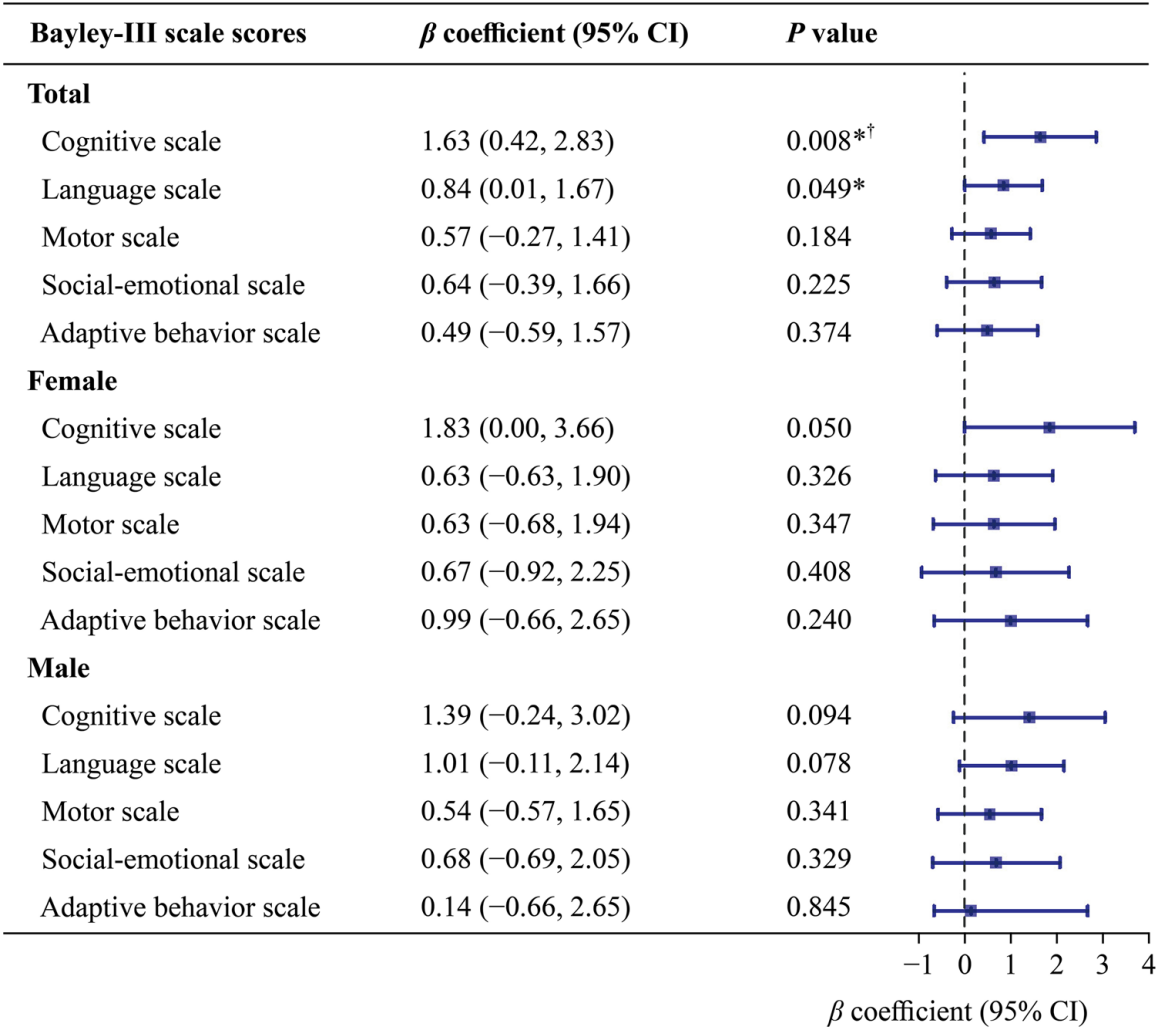
Association of maternal gestational total cardiovascular health scores with total (*n* = 1007), female (*n* = 472) and male (*n* = 535) offspring Balley-III subscale scores at 2–3 years old. Confounders considered in the model were the infant’s sex and birth weight, maternal age, prepregnancy maternal BMI, maternal education level, and maternal mood during gestation including anxiety score, depression score, and pressure score. The error bars indicate 95% CIs. *P value < 0.05, ^†^FDR < 0.05. *BMI* body mass index, *CI* confidence interval, *FDR* false discovery rate, *Bayley-III* Baley scales of infant and toddler development, third edition

As shown in Fig. 1, each one-point higher maternal CVH score significantly increased Balley-III cognitive and language subscale scores by 1.63 [95% confidence interval (CI) 0.42–2.83, *P* = 0.008] and 0.84 (95% CI 0.005–1.67, *P* = 0.049), respectively. After FDR correction, the statistical significance remains in the cognitive subscale score. The sex-stratified analysis in Fig. 1 shows that no significant association was found in female or male offspring although the effect estimates seemed to be more pronounced for female offspring’s cognitive development (*β*_adj_ = 1.83, 95% CI − 0.004–3.66, *P* = 0.050).

Maternal CVH status (ideal-only, intermediate-only, one-poor, or multiple-poor) also influenced neurodevelopment at 2–3 years of age (Supplementary Fig. 3). Compared with those in the ideal-only group, the cognitive and language subscale scores in the intermediate-only, one-poor, and multipoor groups were significantly lower. In particular, for the cognitive subscale scores in the intermediate-only and one-poor groups and the language subscale scores in the intermediate-only group, the significant reduction persisted after FDR correction. An association was not found for the rest of the subscales (Supplementary Fig. 3a). After sex stratification (Supplementary Fig. 3b), maternal CVH status did not affect neurodevelopment in male offspring. In female offspring, cognitive subscale scores were significantly lower in the intermediate-only, one-poor, and multiple-poor groups than in the ideal-only group (persisting after FDR correction). Language subscale scores were significantly lower in the intermediate-only and one-poor groups precorrection, but only the intermediate-only group remained significant after FDR adjustment.

#### Neurodevelopment at 4–5 years of age

**Fig. 2:**
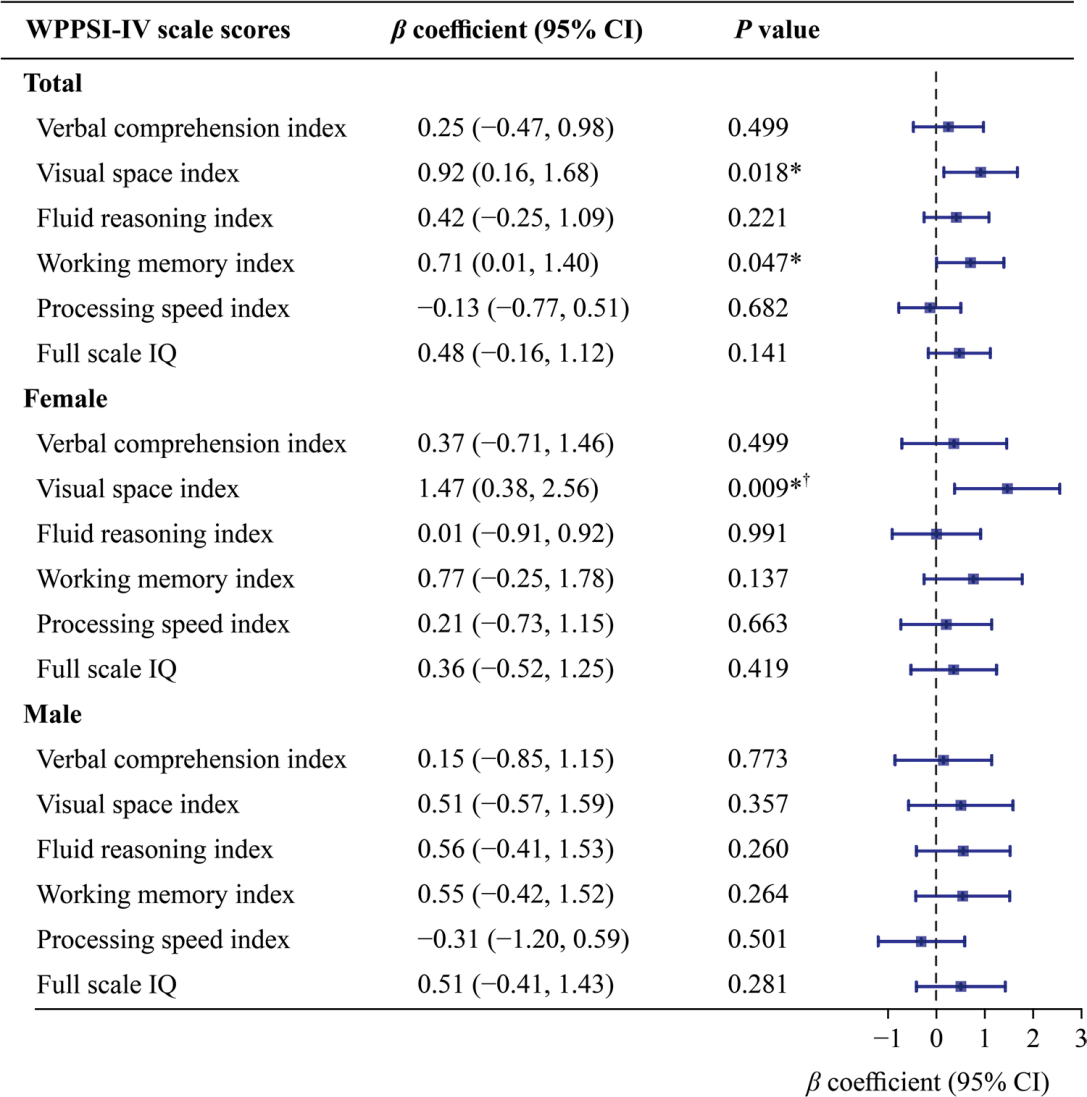
Associations of maternal gestational cardiovascular health scores with total (*n* = 1007), female (*n* = 472) and male (*n* = 535) offspring Wechsler preschool and primary scale of intelligence, fourth edition (WPPSI-IV) subscale scores at 4–5 years old. Confounders considered in the model were the infant’s sex and birth weight, maternal age, prepregnancy maternal BMI, maternal education level, and maternal mood during gestation including anxiety score, depression score, and pressure score. The error bars indicate 95% CIs. *P value < 0.05, ^†^FDR < 0.05. *BMI* body mass index, *CI* confidence interval, *IQ* intelligence quotient

Figure 2 shows that maternal CVH scores were significantly positively associated with VSI (*β*_adj_ = 0.92, 95% CI 0.16–1.68, *P* = 0.018) and WMI (*β*_adj_ = 0.71, 95% CI 0.01–1.40, *P* = 0.047). After sex stratification, maternal CVH scores were significantly positively correlated with VSI scores in female offspring, regardless of FDR adjustment (*β*_adj_ = 1.47, 95% CI 0.38–2.56, *P* = 0.009).

There was no association between maternal CVH categories and each index score in total offspring (Supplementary Fig. 4a). Compared with those in the ideal-only group of female offspring, VCI and PSI scores in the intermediate-only group, PSI scores in the one-poor group, and VSI scores in the multiple-poor group were significantly lower; after FDR correction, only the VSI score decreased significantly in the multiple-poor group (Supplementary Fig. 4b). In male offspring, maternal CVH status significantly influenced PSI scores. Specifically, PSI scores were significantly lower in the intermediate-only, one-poor, and multiple-poor groups than in the ideal-only group, and the significance persisted in the intermediate-only and multiple-poor groups after FDR correction (Supplementary Fig. 4b).

### Combined maternal cardiovascular health metrics and suboptimal neurodevelopment

#### Suboptimal neurodevelopment at 2–3 years of age

**Fig. 3:**
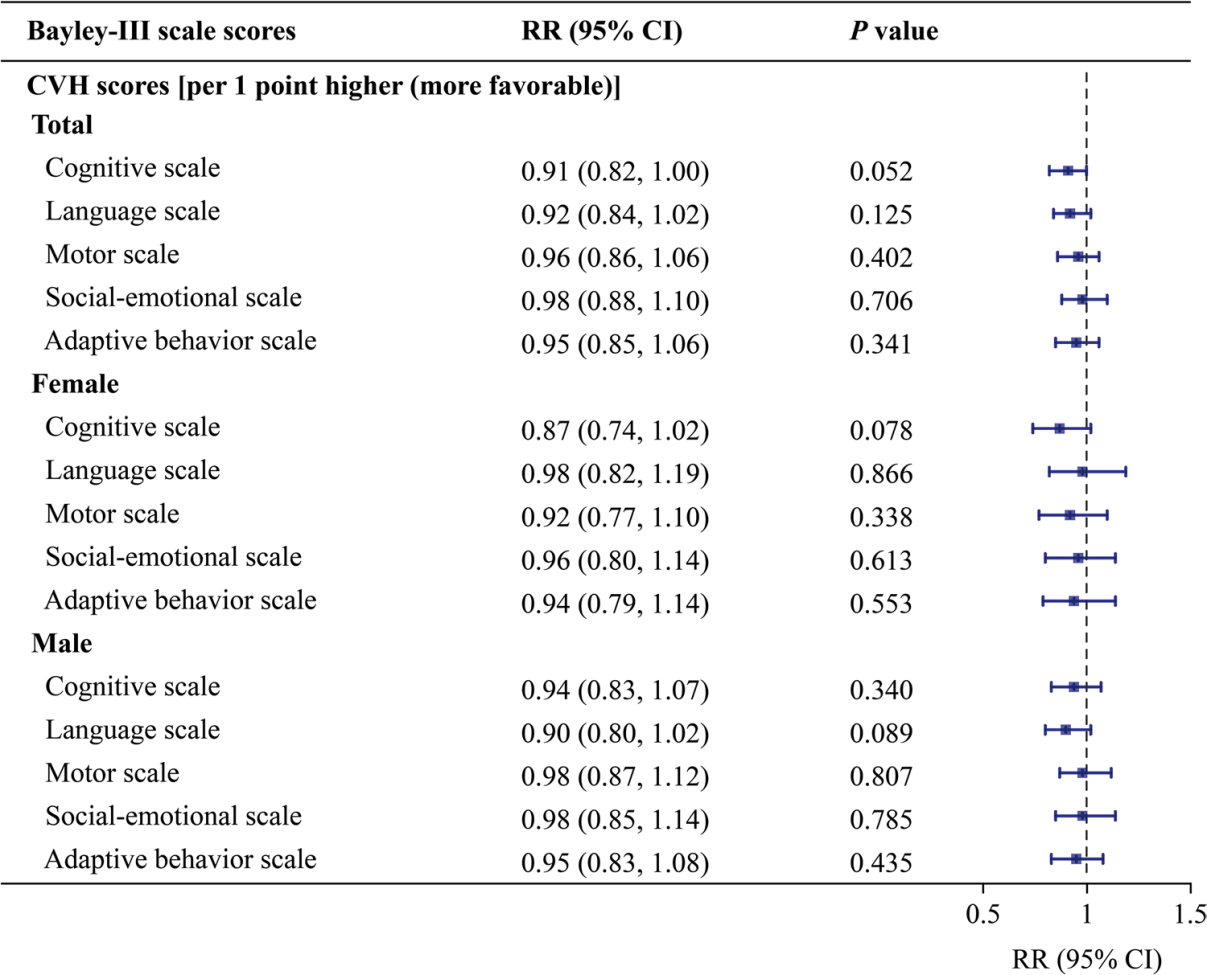
Effect of maternal gestational cardiovascular health on the risk of suboptimal neurodevelopment in total (*n* = 1007), female (*n* = 472) and male offspring (*n* = 535) at 2–3 years old. Confounders considered in the model were the infant’s sex and birth weight, maternal age, prepregnancy maternal BMI, maternal education level, and maternal mood during gestation including anxiety score, depression score, and pressure score. The error bars indicate 95% CIs. *CVH* cardiovascular health, *BMI* body mass index, *CI* confidence interval, *RR* risk ratio, *Bayley-III* Baley scales of infant and toddler development, third edition

According to the Poisson regression model, no significant associations between maternal CVH scores and suboptimal neurodevelopment in offspring were found, regardless of sex (Fig. 3). However, maternal CVH status influenced suboptimal cognitive and language development in offspring at 2–3 years of age (Supplementary Fig. 5). Compared with that in the ideal-only group, the risk of suboptimal cognitive development was significantly greater in all three nonideal groups (intermediate-only, one-poor, and multiple-poor), particularly in the intermediate-only group, which remained significant after FDR adjustment. Comparatively, the risk of suboptimal language development was elevated only in the intermediate-only group, where no significance was preserved following FDR adjustment (Supplementary Fig. 5a). According to the sex-stratified analysis, maternal CVH status had a greater effect on suboptimal cognitive development in female offspring in the intermediate-only and one-poor groups, but with an FDR-adjusted *P* > 0.05 (Supplementary Fig. 5b).

#### Suboptimal neurodevelopment at 4–5 years of age

**Fig. 4:**
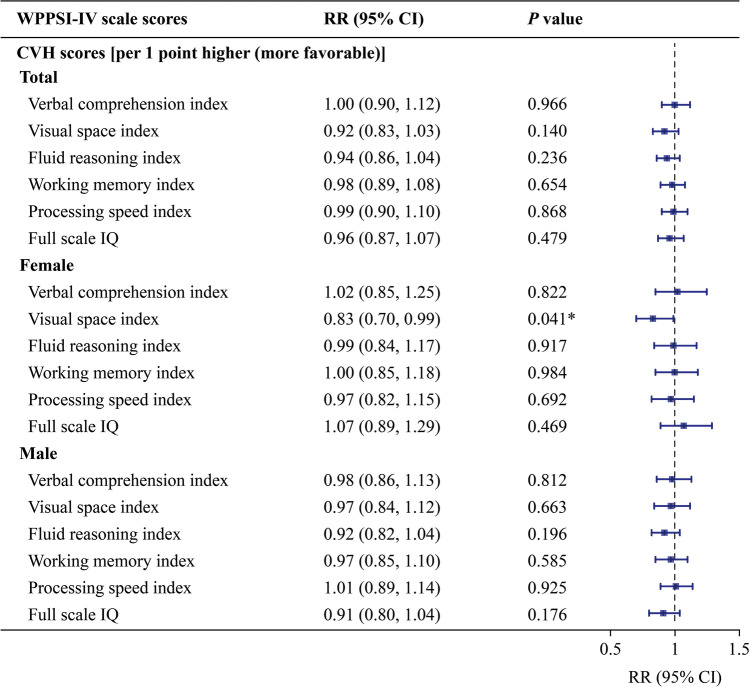
Effect of maternal gestational cardiovascular health on the risk of suboptimal neurodevelopment in total (*n* = 1007), female (*n* = 472) and male offspring (*n* = 535) at 4–5 years old. Confounders considered in the model were the infant’s sex and birth weight, maternal age, prepregnancy maternal BMI, maternal education level, and maternal mood during gestation including anxiety score, depression score, and pressure score. The error bars indicate 95% CIs. *P value < 0.05. *CVH* cardiovascular health, *BMI* body mass index, *CI* confidence interval, *RR* risk ratio, *IQ* intelligence quotient, *WPPSI-IV* Wechsler preschool and primary scale of intelligence, fourth edition

The risk of suboptimal development for each domain in total and male offspring at 4–5 years of age was not affected by a one-point higher gestational CVH score (Fig. 4**)**. In female offspring (Fig. 4), only the risk of suboptimal VSI development was significantly reduced [adjusted relative risk (RR_adj_) = 0.83, 95% CI 0.70–0.99, *P* = 0.041], with an FDR-adjusted *P* > 0.05. Maternal CVH status also showed no overall association with the risk of suboptimal neurodevelopment in 4–5-year-old offspring. Sex-specific subgroup exceptions were observed: females in the multiple-poor group had an elevated risk for suboptimal development in the VSI domain, whereas males in the intermediate-only group presented a decreased risk for suboptimal development in the WMI domain. However, neither association retained statistical significance after FDR adjustment. (Supplementary Fig. 6).

## Discussion

This is a novel prospective cohort study that explored the associations between the combination of maternal cardiovascular health metrics during pregnancy and offspring neurodevelopment in critical periods and revealed that improved maternal CVH positively influences offspring’s cognitive and language development at 2–3 years of age and visual space and working memory function at 4–5 years of age, particularly cognitive development and visual space function in female offspring.

Investigating the impact of combined maternal CVH metric exposure on offspring neurodevelopment would provide more accurate, scientific, and profound findings than single maternal CVH metric exposure. Many studies have revealed the potential mechanisms underlying the associations between poor maternal blood glucose, pressure, and lipids, or sleep deprivation, obesity, nicotine exposure, poor diet, and a lack of physical activity during pregnancy with offspring neurodevelopment. Notably, many underlying mechanisms mediated by these metrics are similar, such as microglial activation and chronic inflammatory responses in the brains of offspring, suboptimal nutrient and oxygen availability for the fetus attributable to impaired placental function, disrupted fetal epigenetic regulation in the brain, etc. [35–39]. Specifically, the combined effect is not simply additive; synergistic interactions likely occur among these factors [40, 41]. Conditions, such as dysglycemia, hypertension, and obesity, commonly cooccur in individuals and interact synergistically [16]. Moreover, combined CVH metrics at a suboptimal level are more prevalent than single CVH metrics are [20]. In our study, the frequency of combined CVH metrics at a suboptimal level in pregnant women was 91.8%, whereas the figures for the separated metrics ranged from 0.1% to 31.2%, except for blood pressure, which had a frequency of 82%.

To date, only one study (mentioned in the “Introduction” section) has investigated the impact of combined maternal CVH metrics on offspring neurodevelopment [16], whereas the previous study assessed neurodevelopment solely at 12 months of age. Our study evaluated neurodevelopment at two time points, extending follow-up through the crucial preschool period (4–5 years old). Presently, the impact of combined maternal gestational CVH metrics on offspring development or cardiovascular health has been widely studied [20, 42]. Although our findings indicate that combined maternal CVH metrics are related to offspring neurodevelopmental delay, the associations between them require confirmation in larger, multicenter studies.

In agreement with the findings of the previous three studies, the current study also demonstrated the associations of the cognitive and language domains in 2–3-year-olds assessed with the Bayley-III and visual–spatial and working memory domains in 4–5-year-olds evaluated with the WPPIS-IV. Månsson et al. [43] found that preterm infants’ cognitive and language subscale scores assessed with the Bayley-III at a corrected age of 2.5 years were correlated with working memory index development measured with the WISC-IV at 6.5 years of age. Similarly, Flynn et al. [34] reported that preterm infants’ Bayley-III or Bayley scales of infant and toddler development, second edition (BSID-II) language scores at 19–21 months were positively correlated with working memory index scores at 47–58 months. Kalstabakken et al. [44] found that Bayley-III cognitive and language subscale scores in children at one and two years of age, particularly two years of age, were significantly and positively correlated with the WPPSI-IV VSI and WMI scores at three years of age. In general, visual–spatial and working memory skills are parts of cognitive development and are affected by early language development. Studies have confirmed that children with language impairment have greater difficulty in visuospatial tasks, indicating a strong relationship between the two domains [45]. Generally, working memory and language are integrated and intertwined, and better language facilitates the development of working memory, which in turn enhances language acquisition and processing [46].

In our study, combined maternal CVH exposure affected mainly the cognitive, language, visual space, and working memory domains in offspring within the first five years of life. With respect to single maternal CVH metric exposure, previous studies similarly indicated that these domains in offspring are affected. School-aged children born to mothers with gestational diabetes presented low spatial skills and lower scores for the general intellectual level and working memory index [47]. Similarly, reduced scores on the general cognitive scale and memory span have also been reported in preschool children whose maternal sleep duration is less than eight hours in late pregnancy [9], and more cognitive problems have been reported in 4.5-year-old children born to mothers with early and severe hypertensive complications during pregnancy [48]. One study reported that children born to obese mothers experienced temporarily accelerated development of cognition and language, followed by rapid deceleration until 18 months of age, particularly in terms of language scores [49]. In sensitive windows, the affected domains in offspring exposed to other risk factors during pregnancy are almost the same [50]. In general, among the visual, auditory, language and cognitive function development related to the above four domains, the former three almost finish within the first five years of life; the latter continues to early adulthood, but the peak occurs around 1–3 years of age [51]. Therefore, focusing on offspring neurodevelopment to five years of age in our study will better predict adolescents’ or adults’ neurodevelopment or help establish new strategies for early intervention in potential neurodevelopmental delay or damage.

Our research demonstrated that higher maternal CVH scores are likely to confer greater neurodevelopmental benefits to female offspring than to male offspring. Some maternal CVH metrics have also been shown to exert sex-specific influences on offspring neurodevelopment, although findings remain inconsistent. One review reported that female offspring of obese mothers were more prone to anxiety than male offspring were in human studies [52]. In a clinical study, researchers reported that prepregnancy obesity was associated with lower psychomotor development index scores among boys, but not among girls, aged three years [53]. Similarly, one prospective study indicated that maternal prepregnancy overweight and obesity are associated with a reduction of 5.7–7.1 intelligence quotient (IQ) points only among boys [53]. Animal studies involving single poor maternal CVH metric exposure have shown that neurodevelopment impairments are more likely to occur in female offspring. One rat experiment revealed that the female offspring of diabetic dams exhibited suboptimal cognitive abilities [54]. In a mouse model, maternal obesity increased anxiety and decreased sociability in female offspring but not in male offspring [55]. Owing to gonadal hormonal exposure during fetal and postnatal life, which confers different susceptibilities to genetic and environmental exposures, sex indeed influences the neurodevelopmental trajectory, as well as the clinical presentation, biology, and treatment response [56]. In our study, the maternal CVH measure lacked three metrics, and further research is needed to determine whether the observed sex-specific disparity in offspring neurodevelopment persists with a full eight-metric composite.

The first 1000 days of life is key to lifelong health and well-being [57], and many studies have revealed the effects of CVH exposure during different trimesters on neurodevelopment. For example, in the Jiangsu Birth Cohort Study, Lv et al. [58] examined the associations between maternal dietary patterns at mid- and late gestation and infants’ neurodevelopment at 12 months. Their results revealed that the maternal “aquatic products, fresh vegetables and Homonemeae” pattern in the second trimester was associated with a lower risk of being noncompetent in terms of cognitive and gross motor development, and a similar dietary pattern in the third trimester was also significantly associated with a decreased risk of failing age-appreciate cognitive and receptive communication development. Polanska et al. [59] assessed the impact of environmental tobacco smoke (ETS) exposure during three trimesters of pregnancy on child neurodevelopment (BSID evaluation) within the first two years of life. The results showed that ETS exposure in the first and second trimesters was associated with decreasing child language function at the age of one and two years; a negative association was found for 1.5 ng/mL cotinine in the second trimester and child cognition at two years, as well as 1.5 ng/mL cotinine in all trimesters and child motor abilities at two years. Domingues et al. [13] reported a positive association between leisure-time physical activity practices during any trimester and offspring neurodevelopment at 12 months. In our study, only maternal CVH exposure in the second trimester was available. To better understand the critical time window for the impact of CVH metric exposure, particularly the combination of all CVH metric exposures, on offspring neurodevelopment, larger, prospective, multicenter, population-based studies involving exposure stages, including the three trimesters or early days after birth, are needed in future.

To date, this is an updated study to reveal the influence of a combination of maternal gestational CVH levels on offspring brain development at two time points and to confirm the enduring effect of maternal CVH exposure on offspring neurodevelopment within the critical period—the first five years of life. However, some limitations undermine the quality of our findings. First, since information on total cholesterol level, diet, and physical activity was absent from the questionnaire, the maternal gestational CVH in our study was a partial CVH. Second, the CVH metric data were available only in the second trimester, and the outcomes of offspring neurodevelopment after CVH exposure throughout pregnancy require further investigation. Third, although multiple confounders were considered in our study, we could not exclude the possibility of confounding from other factors, such as genetic factors, feeding patterns, or early exposure to antibiotics [60].

In conclusion, the present study demonstrated that poor maternal gestational CVH exposure is a risk factor for offspring neurodevelopment, with a significant impact on offspring cognitive, language, visual–spatial, and working memory domain development within crucial periods. Currently, the frequency of suboptimal levels of CVH in pregnant women is high and continues to increase due to unhealthy diets and behaviors. However, the impact of suboptimal levels of maternal CVH on offspring neurodevelopment has rarely been studied. In future, population-based prospective cohort studies comprehensively assessing the exposure of all eight maternal CVH metrics are warranted.

## Supplementary Information

Below is the link to the electronic supplementary material.

Supplementary file1 (DOCX 2492 KB)

## Data Availability

The data are available from the corresponding author on reasonable request.
