# Supplementary material for: Maternal cardiovascular health and offspring neurodevelopment within the first five years of life: a birth cohort study
Source: World J Pediatr. 2025 Sep 19;21(10):991–1002. doi: 10.1007/s12519-025-00969-5 (PMC12578739; doi:10.1007/s12519-025-00969-5)
Supplement: Supplementary file 1 — Supplementary file1 (DOCX 2492 KB) [file 12519_2025_969_MOESM1_ESM.docx]

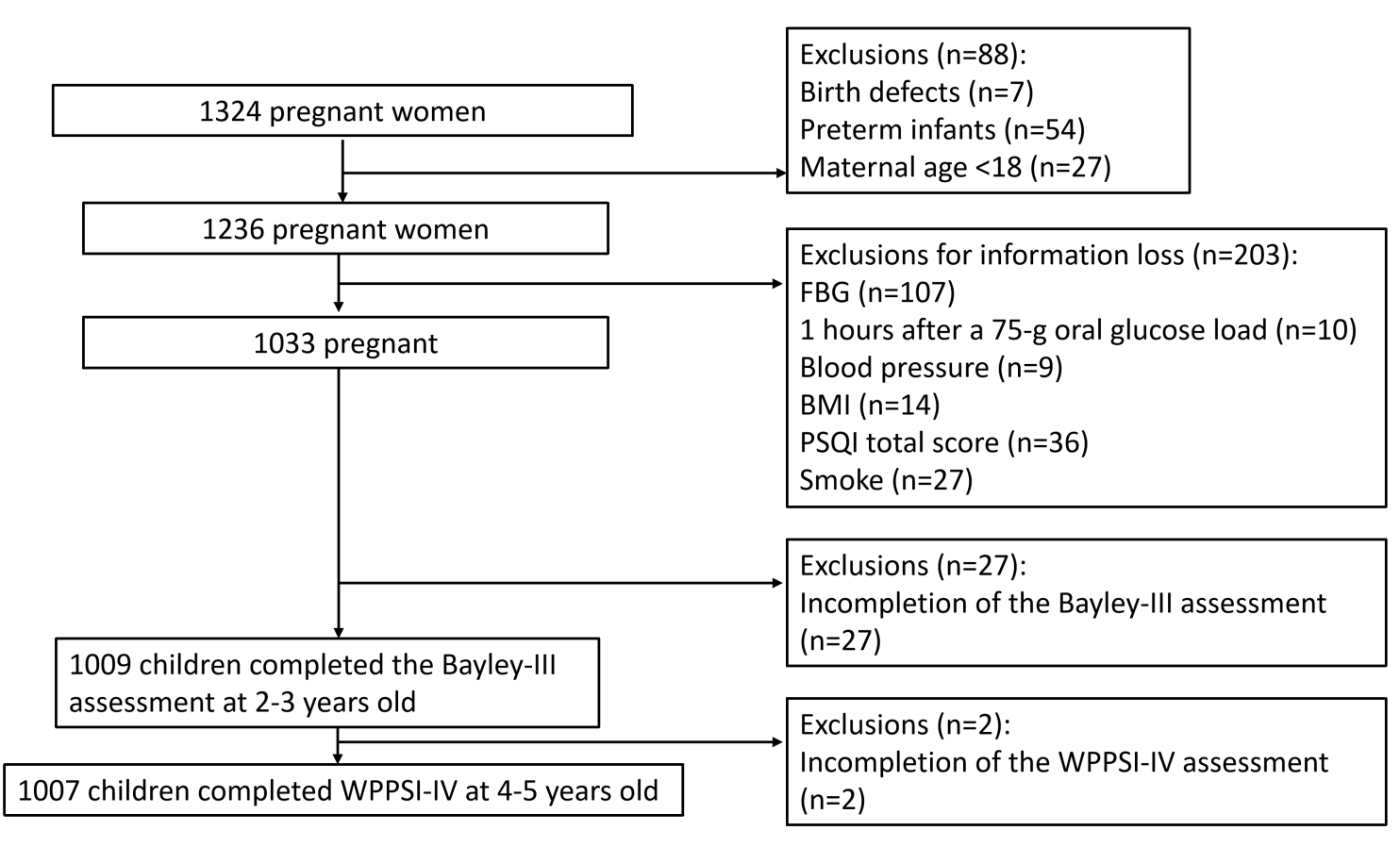


**Fig1**: Flow chart of the participants included in the study.


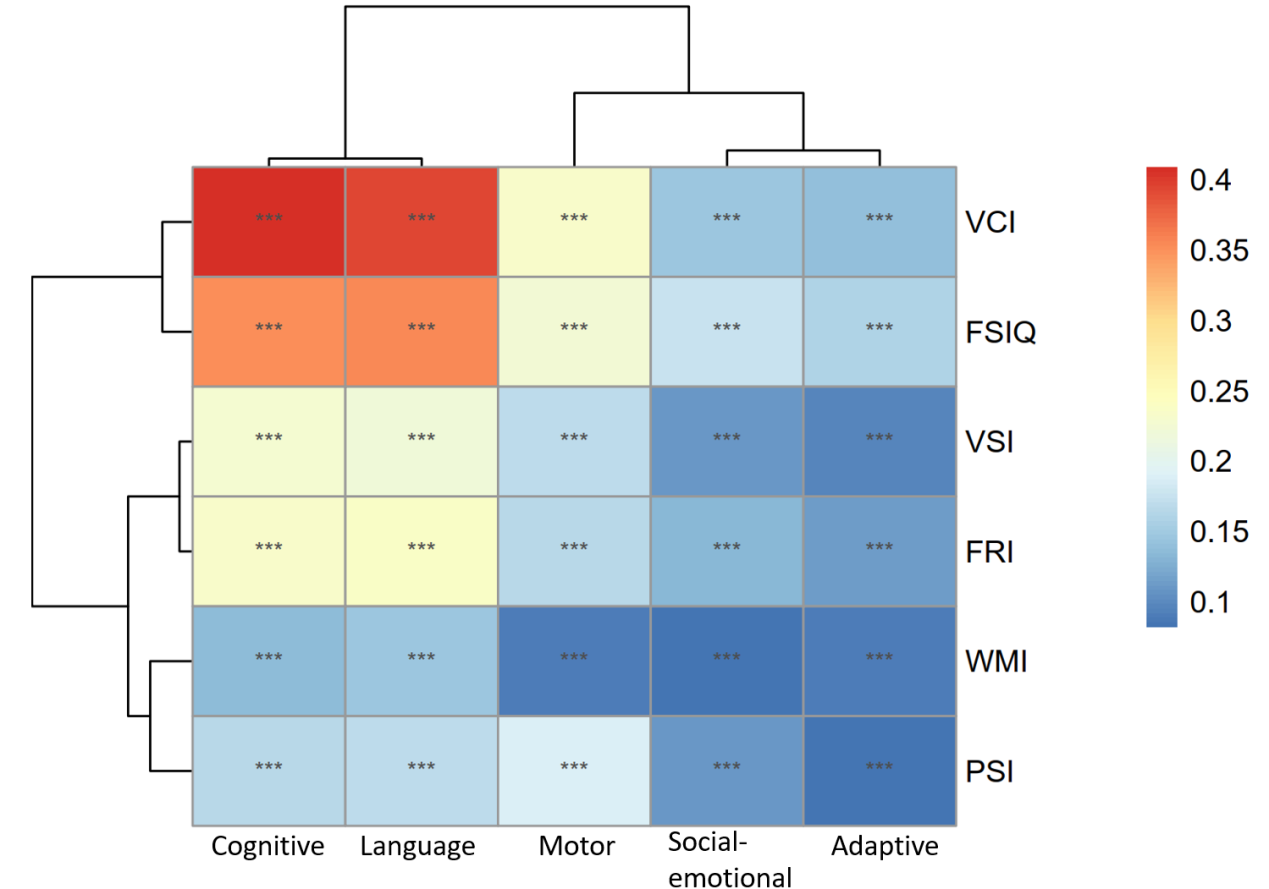


**Fig.2** The correlation analysis of each domain of Bayley-III at 2-3 years old and each index of WPPSI-IV including FSIQ at 4-5 years old. ***P<0.001

a


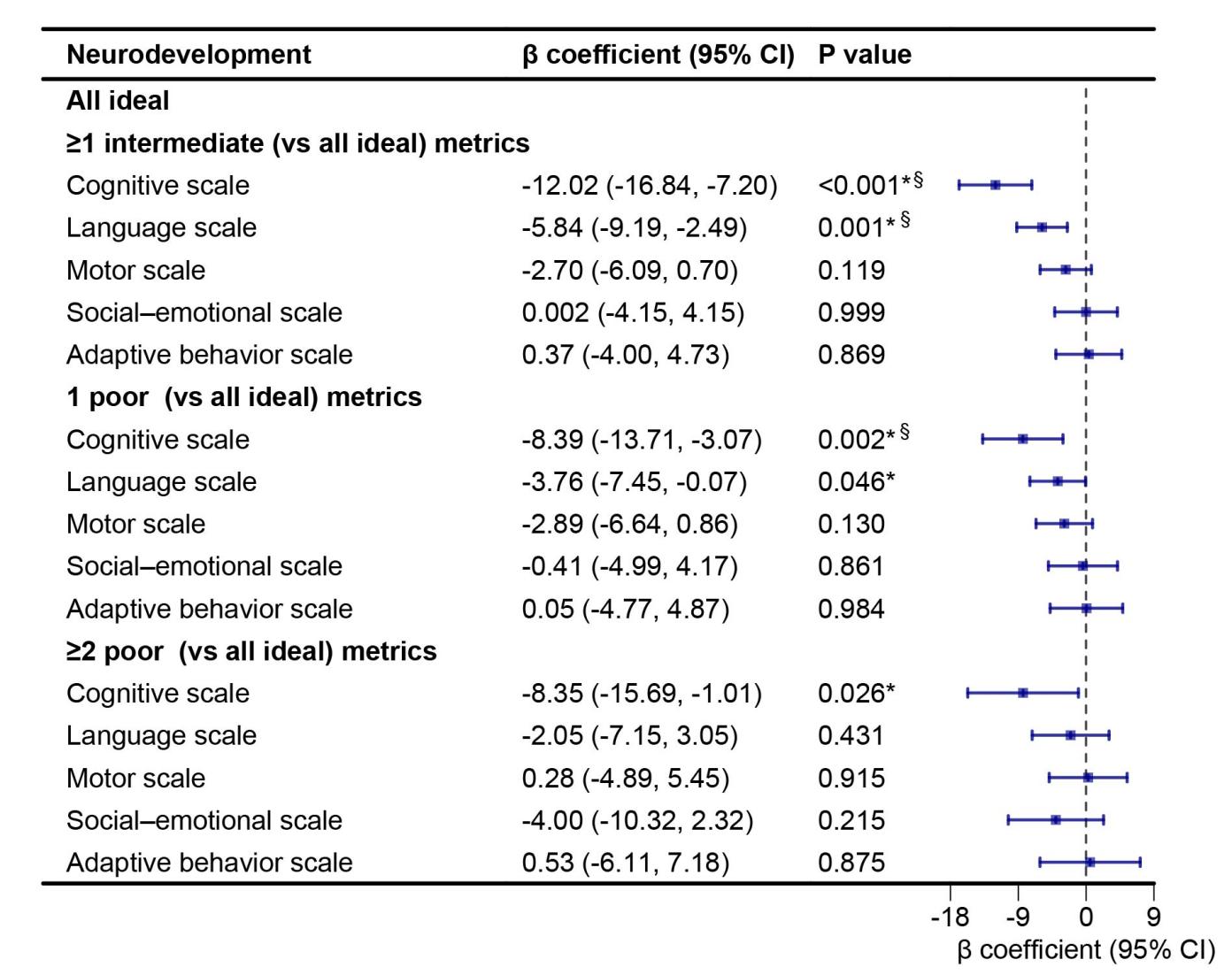


b


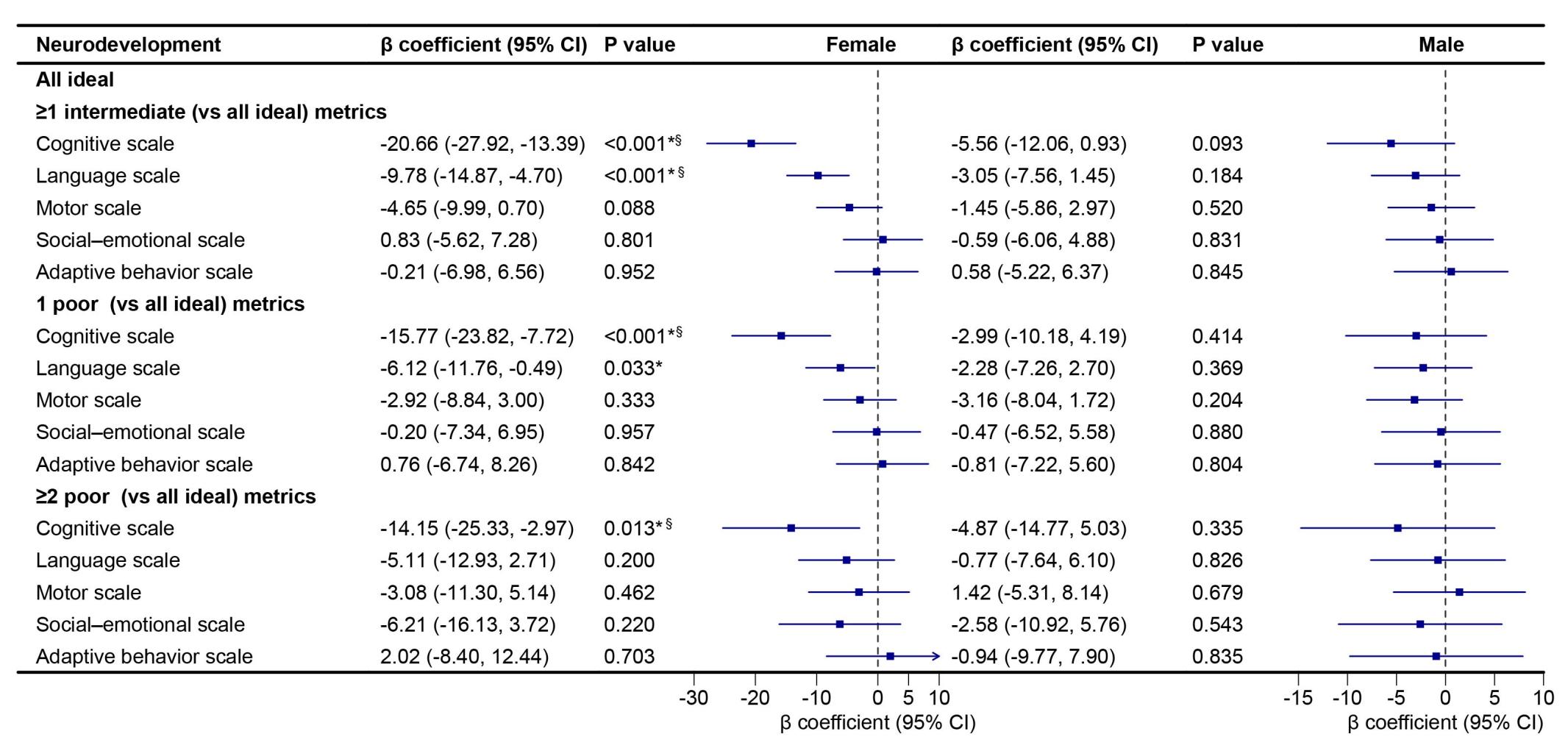


**Fig. 3** The association of maternal CVH levels and total (n=1007) (a), female (n=472) and male (n=535) offspring (b) Bayley-III subscale scores. The confounders: infant’s sex, birth weight, maternal age, pre-pregnancy maternal BMI, maternal education level, and maternal mood during gestation including anxiety score, depression score, and pressure score. The error bars indicate 95% CIs. **P* value <0.05. ^§^ FDR< 0.05. *CI* confidence interval.

a


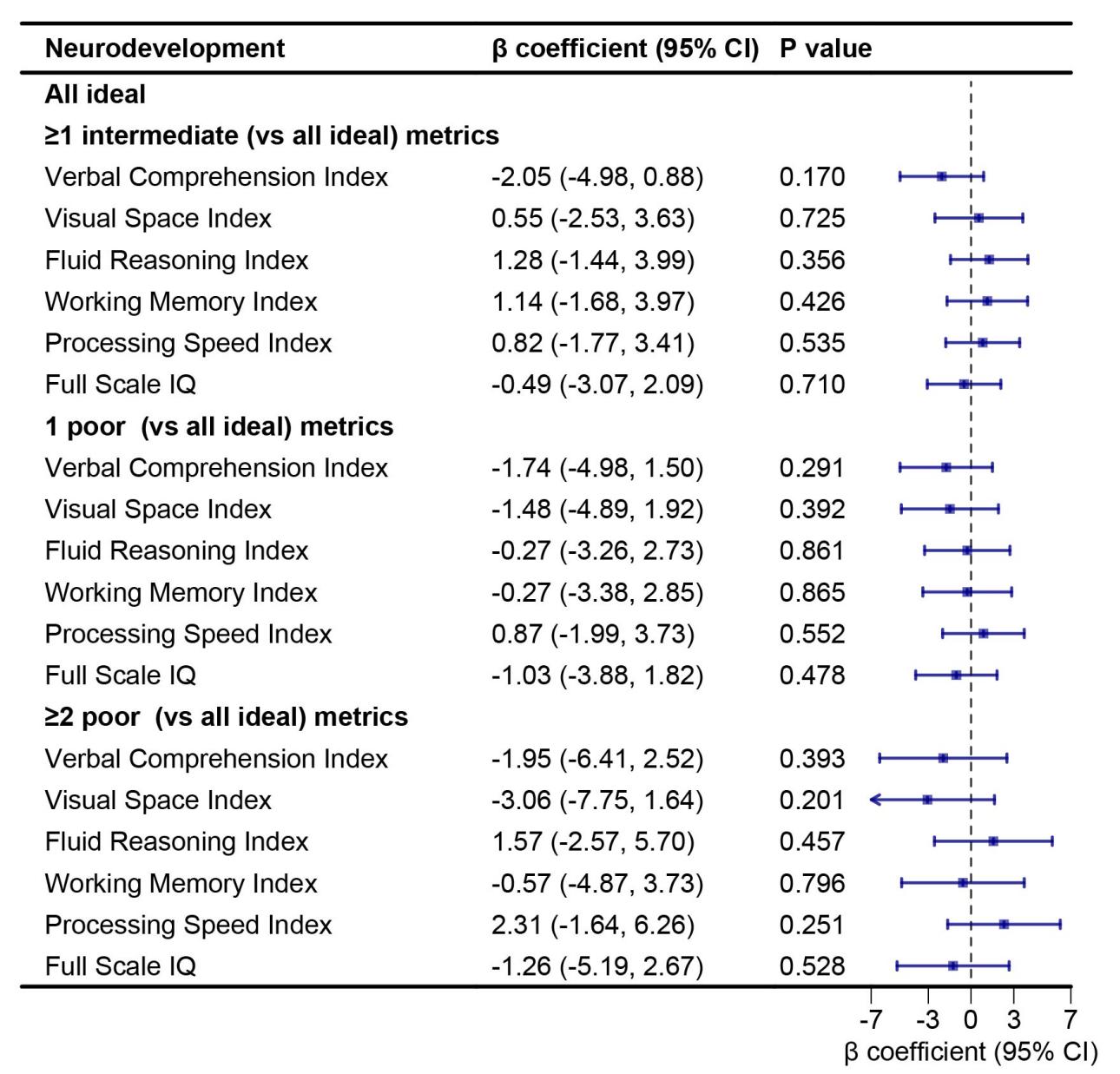


b


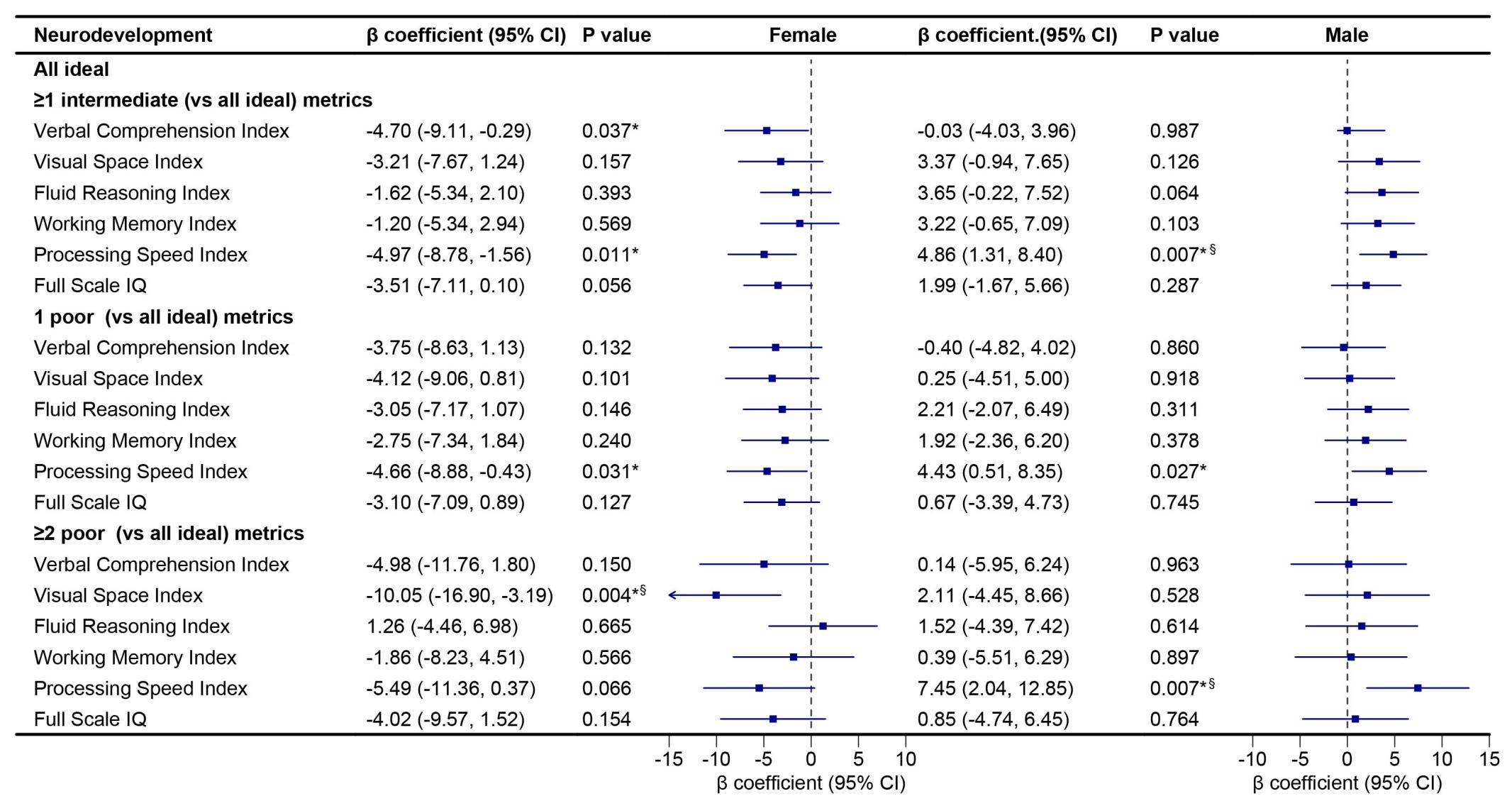


**Fig.4** The association of maternal CVH levels and total (a), female (N=472) and male (N=535) (b) offspring WPPSI-IV scores. The confounders: infant’s sex, birth weight, maternal age, pre-pregnancy maternal BMI, maternal education level, and maternal mood during gestation including anxiety score, depression score, and pressure score. The error bars indicate 95% CIs. **P* value <0.05. ^§^ FDR< 0.05. *CI* confidence interval.

a


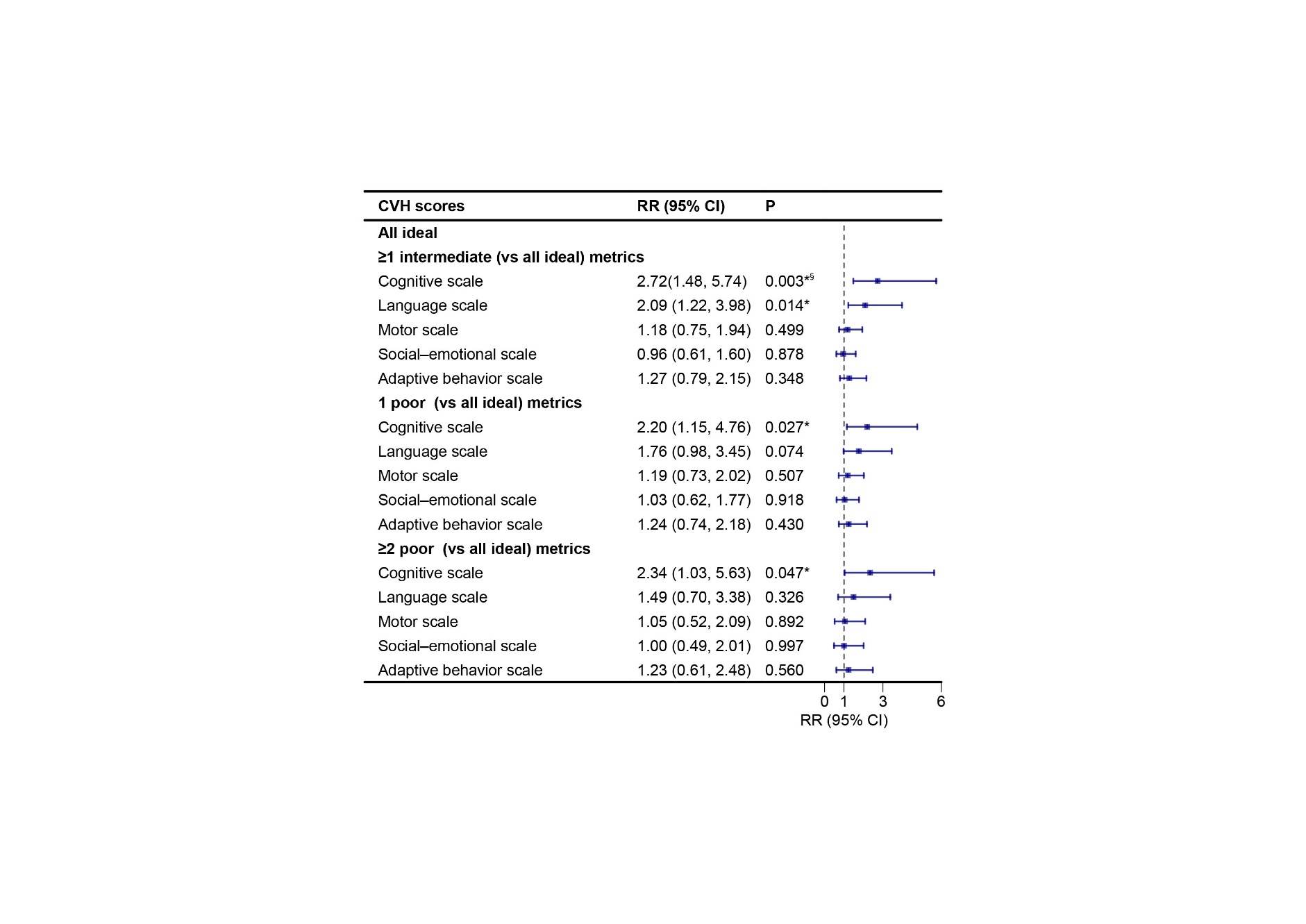


b


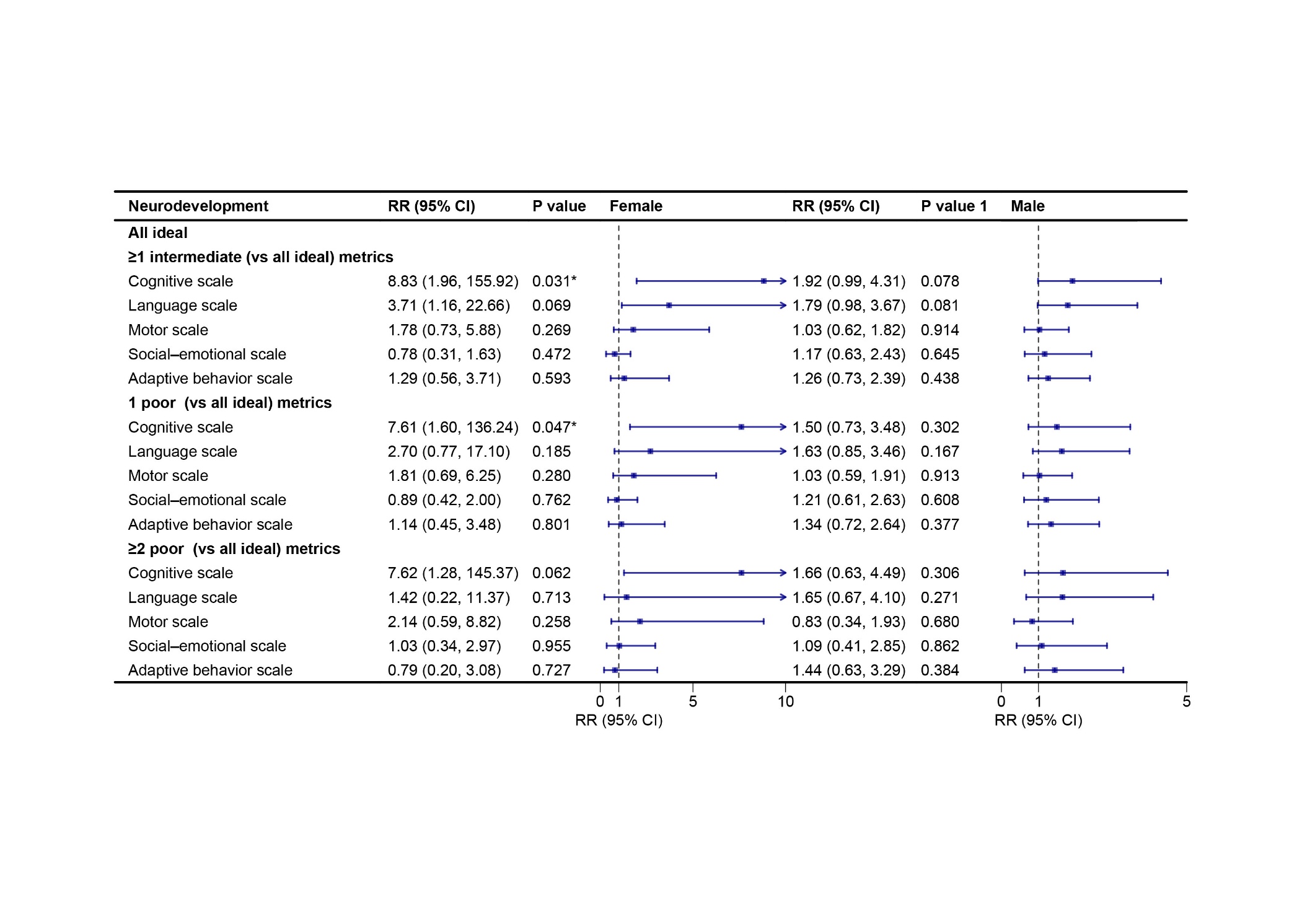


**Fig 5** Effect of maternal CVH status on the risk of suboptimal neurodevelopment in total (n=1007) (a), female (n=472) and male offspring (n=535) (b) at 24 months. The confounders: infant’s sex, birth weight, maternal age, pre-pregnancy maternal BMI, maternal education level, and maternal mood during gestation including anxiety score, depression score, and pressure score. The error bars indicate 95% CIs. **P* value <0.05. ^§^ FDR< 0.05. *CI* confidence interval, *RR* risk ratio

a


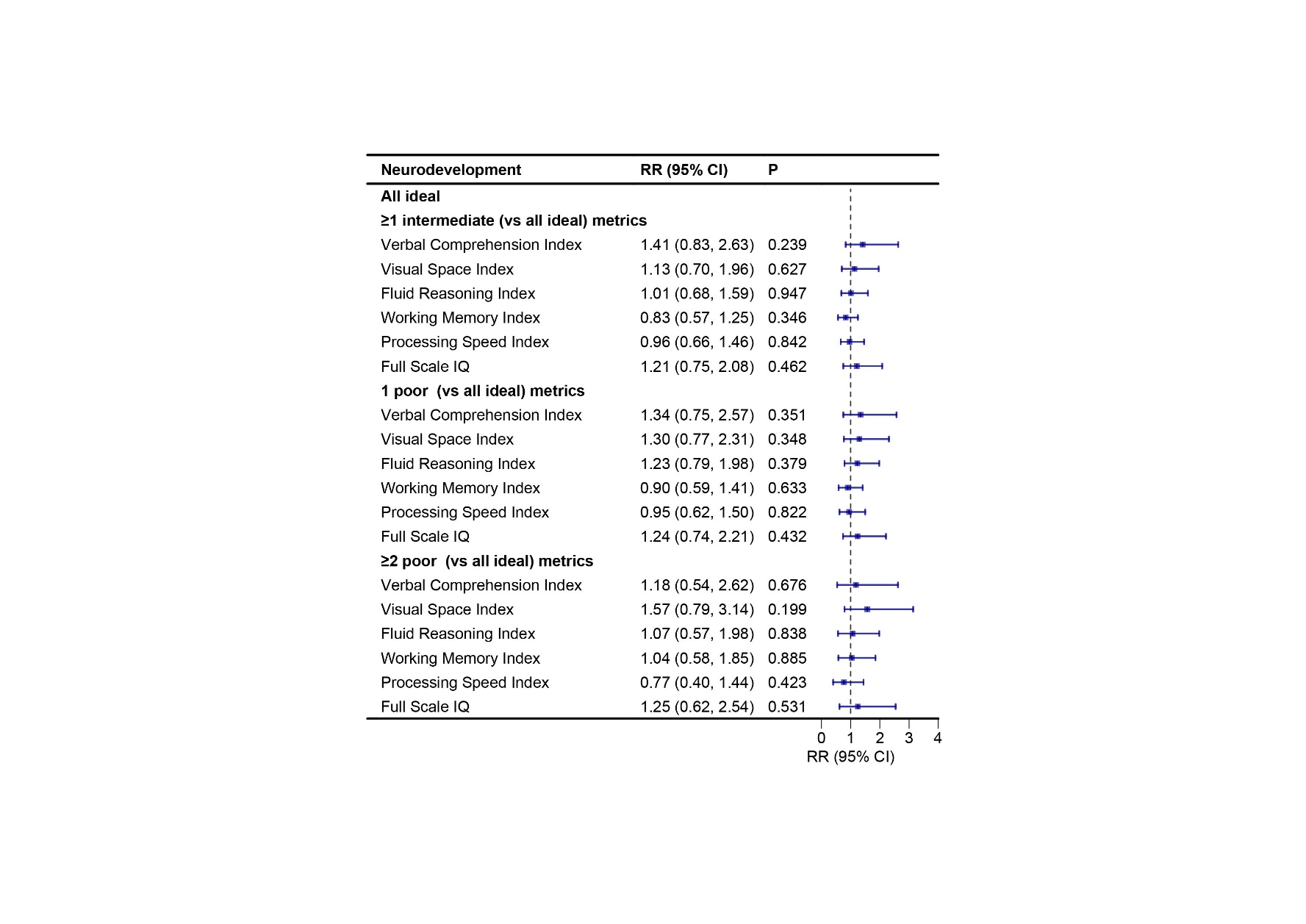


b


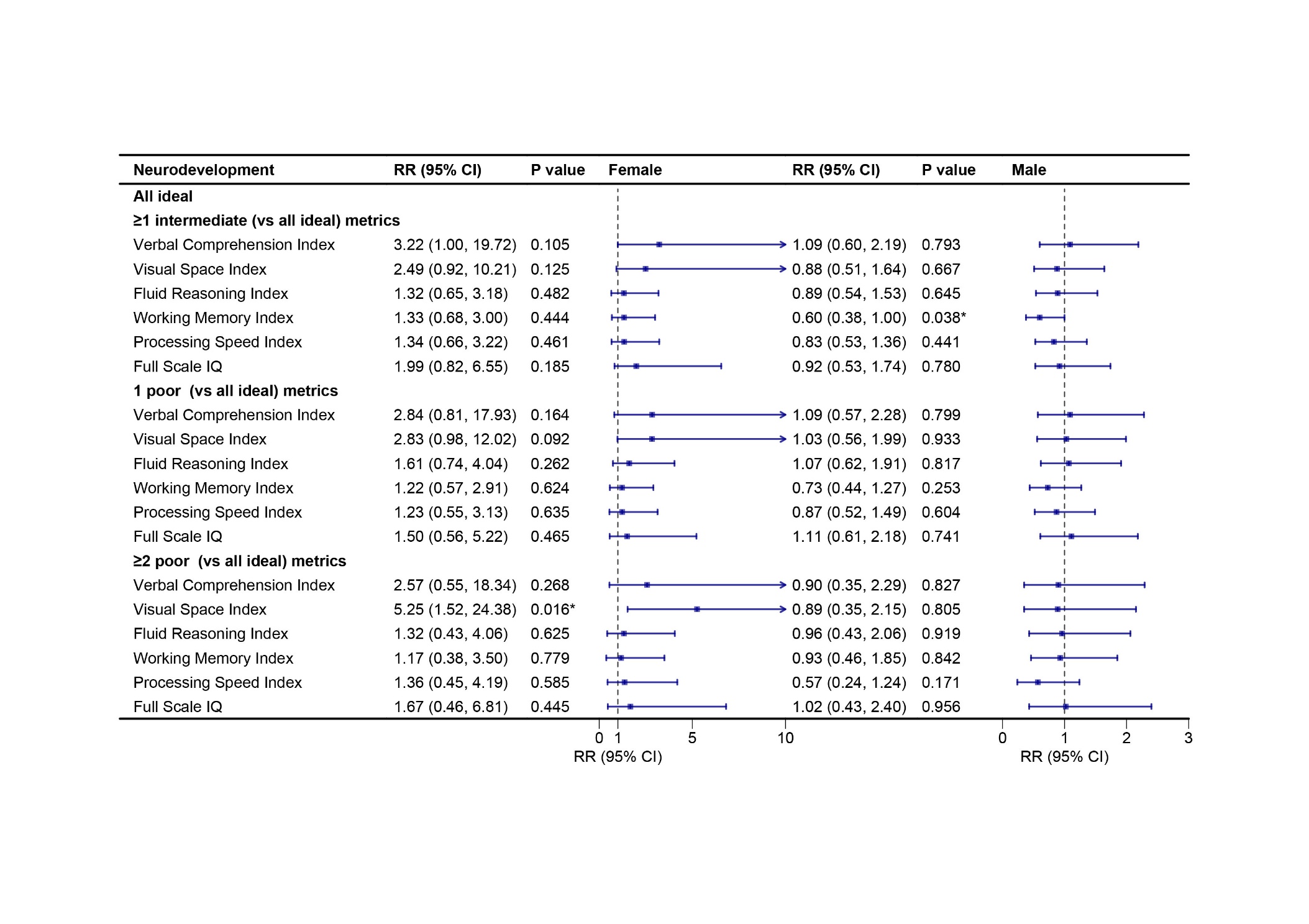


**sFig. 6** The effect of maternal CVH status on the risk of suboptimal neurodevelopment in total (n=1007) (A), female (n=472) and male offspring (n=535) (B) at 2-3 years old. The confounders: infant’s sex, birth weight, maternal age, pre-pregnancy maternal BMI, maternal education level, and maternal mood during gestation including anxiety score, depression score, and pressure score. The error bars indicate 95% CIs. **P* value <0.05. *CI* confidence interval, *RR* risk ratio

Table1: Classification of Cardiovascular Health Metrics for Mother.

|  | Source | Ideal metrics  (2 points) | Intermediate metrics (1 point) | Poor metrics  (0 points) |
| --- | --- | --- | --- | --- |
| BMI (kg/m^2^) | the WHO classification of obesity | ≤28.4 | 28.5-32.9 | ≥33 |
| Blood glucose (mmol/L) | International Association of Diabetes and Pregnancy Study Groups | All others | / | Gestational diabetes: FBG≥5.1 or 1-h OGTT≥10.0 or 2-h OGTT≥8.5 |
| Blood pressure (mmHg) | Chinese expert consensus on blood pressure management during pregnancy | SBP<120 and DBP<80 | SBP 120-139 or DBP 80-89 | SBP≥140 or DBP≥90 |
| PSQI total score |  | ≤5 | 6-10 | ≥11 |
| Smoke |  | No smoke | / | Active smoking |

*BMI* body mass index, *FBG* fasting blood glucose, *OGTT* oral glucose tolerance test, *SBP* systolic blood pressure, *DBP* diastolic blood pressure, *PSQI* Pittsburgh Sleep Quality Index.

Table 2: Offspring neurodevelopment scores by maternal gestational CVH levels (n = 1007).

|  | All ideal metrics | ≥1 intermediate (0 poor) metrics | 1 poor metrics | ≥2 poor metrics | Overall |
| --- | --- | --- | --- | --- | --- |
| Neurodevelopment | Mean ± SD | Mean ± SD | Mean ± SD | Mean ± SD | Mean ± SD |
| Number | 83 | 601 | 264 | 59 | 1007 |
| Bayley-III scales |  |  |  |  |  |
| Cognitive scale | 126 ± 20.2 | 110 ± 22.2 | 113 ± 23.2 | 115 ± 22.6 | 113 ± 22.7 |
| Language scale | 102 ± 15.6 | 94.4 ± 15.1 | 95.9 ± 15.7 | 98.2 ± 16.0 | 95.6 ± 15.5 |
| Motor scale | 110 ± 16.1 | 107 ± 14.8 | 107 ± 14.3 | 111 ± 17.4 | 107 ± 5.0 |
| Social–emotional scale | 106 ± 17.7 | 105 ± 18.1 | 104 ± 18.2 | 101 ± 18.0 | 105 ± 18.1 |
| Adaptive behavior scale | 104 ± 17.3 | 106 ± 19.4 | 105 ± 18.8 | 103 ± 19.7 | 105 ± 19.1 |
| WPPSI-IV scales |  |  |  |  |  |
| Verbal Comprehension Index | 119 ± 14.0 | 115 ± 13.2 | 114 ± 14.2 | 113 ± 14.5 | 115 ± 13.6 |
| Visual Space Index | 115 ± 14.2 | 114 ± 13.8 | 112 ± 13.0 | 110 ± 12.6 | 113 ± 13.6 |
| Fluid Reasoning Index | 110 ± 14.0 | 110 ± 11.5 | 108 ± 12.7 | 110 ± 13.0 | 110 ± 12.1 |
| Working Memory Index | 105 ± 14.4 | 106 ± 12.3 | 104 ± 11.1 | 103 ± 13.2 | 105 ± 12.3 |
| Processing Speed Index | 105 ± 17.4 | 106 ± 10.6 | 106 ± 10.8 | 106 ± 10.7 | 106 ± 11.4 |
| Full Scale IQ | 117 ± 13.3 | 115 ± 11.9 | 113 ± 11.2 | 113 ± 11.8 | 114 ± 11.9 |

*SD* standard deviation, *WPPSI-IV* Wechsler preschool and primary scale of intelligence, fourth edition, *IQ* intelligence quotient.

Table 3: Associations of the characteristics of mother-infant pairs with Bayley-III subscale scores at 2-3 years old.

| Characteristics | Cognitive scale | | Language scale | | Motor scale | | Social–emotional scale | | Adaptive behavior scale | |
| --- | --- | --- | --- | --- | --- | --- | --- | --- | --- | --- |
|  | *β* coefficient (95% CI) | *P* value | *β* coefficient  (95% CI) | *P* value | *β* coefficient  (95% CI) | *P* value | *β* coefficient  (95% CI) | *P* value | *β* coefficient (95% CI) | *P* value |
| **Exposure factor** |  |  |  |  |  |  |  |  |  |  |
| BMI (kg/m^2^) | -0.36  (-0.80, 0.07) | 0.10 | -0.18  (-0.48, 0.12) | 0.23 | 0.04  (-0.25, 0.33) | 0.78 | 0.02  (-0.33, 0.37) | 0.92 | -0.24  (-0.61, 0.13) | 0.20 |
| Blood glucose (mmol/L) |  |  |  |  |  |  |  |  |  |  |
| FBG | -5.77  (-8.73, -2.81) | 0.00* | -2.77  (-4.79, -0.74) | 0.007* | -1.12  (-3.09, 0.84) | 0.26 | -1.36  (-3.73, 1.00) | 0.26 | -1.46  (-3.96, 1.05) | 0.25 |
| 1 hour after a 75-g oral glucose load | 0.92  (0.09, 1.76) | 0.03* | 0.54  (-0.03, 1.11) | 0.06 | 0.40  (-0.15, 0.95) | 0.16 | -0.46  (-1.13, 0.20) | 0.17 | -0.22  (-0.92, 0.48) | 0.52 |
| 2 hours after a 75-g oral glucose load | -0.11  (-1.09, 0.88) | 0.83 | -0.32  ( -0.98, 0.35) | 0.35 | -0.05  (-0.70, 0.60) | 0.88 | -0.13  (-0.91, 0.65) | 0.74 | 0.08  (-0.74, 0.91) | 0.85 |
| Blood pressure (mmHg) |  |  |  |  |  |  |  |  |  |  |
| systolic pressure | 0.10  (-0.04, 0.23) | 0.16 | 0.11  (0.01, 0.20) | 0.02* | 0.11  (0.02, 0.20) | 0.01* | 0.00  (-0.11, 0.11) | 0.98 | -0.09  (-0.20, 0.02) | 0.12 |
| diastolic pressure | -0.14  (-0.34, 0.05) | 0.16 | -0.09  (-0.22, -0.04) | 0.19 | -0.09  (-0.22, -0.04) | 0.17 | -0.15  (-0.31, -0.00) | 0.05 | -0.09  (-0.24, 0.08) | 0.35 |
| PSQI total score | -1.15  (-1.71, -0.58) | 0.00* | -0.90  (-1.28, -0.52) | 0.00* | -0.60  (-0.98, -0.23) | 0.002* | -0.20  (-0.66, 0.25) | 0.38 | -0.37  (-0.85, 0.11) | 0.13 |
| smoke |  |  |  |  |  |  |  |  |  |  |
| No smoke | / | / | / | / | / | / | / | / | / | / |
| active smoking | 32.32  (-12.26, 76.89) | 0.16 | 4.39  (-26.00, 34.78) | 0.78 | -10.20  (-39.64, 19.24) | 0.50 | -14.54  (-50.00, 20.93) | 0.42 | 14.67  (-22.79, 52.14) | 0.44 |
| **Basic information** |  |  |  |  |  |  |  |  |  |  |
| Maternal age (y) | 1.51  (1.13, 1.88) | 0.00* | 0.68  (0.42, 0.94) | 0.00* | 0.59  (0.34, 0.83) | 0.000* | 0.17  ( -0.12, 0.49) | 0.23 | -0.68  (-0.99, -0.36) | 0.00* |
| Pre-pregnant BMI (kg/m^2^) | 0.08  (-0.38, 0.53) | 0.75 | 0.04  (-0.27, 0.35) | 0.79 | 0.21  (-0.09, 0.51) | 0.16 | -0.03  (-0.39, 0.33) | 0.87 | -0.31  (-0.70, 0.07) | 0.11 |
| Birth weight (g) | -0.002  (-0.005, 0.001) | 0.16 | -0.003  (-0.005, -0.0003) | 0.02* | 0.00  (-0.003, 0.002) | 0.60 | 0.00  (-0.002, 0.003) | 0.70 | 0.002  (-0.0008, 0.005) | 0.16 |
| infant gender |  |  |  |  |  |  |  |  |  |  |
| male | / | / | / | / | / | / | / | / | / | / |
| female | 4.25  (1.46, 7.05) | 0.003* | 5.08  (3.20, 6.97) | 0.00* | 4.01  (2.18, 5.84) | 0.00* | 2.23  (0.01, 4.46) | 0.05 | 6.31  (3.98, 8.63) | 0.00* |
| Maternal education |  |  |  |  |  |  |  |  |  |  |
| Below high school degree | / | / | / | / | / | / | / | / | / | / |
| High school degree | -74  (-10.32, 8.84) | 0.88 | 2.95  (-3.64, 9.55) | 0.38 | 1.73  (-4.91, 8.36) | 0.61 | 1.64  (-6.47, 9.75) | 0.69 | 0.50  (-8.11, 9.10) | 0.91 |
| College degree | 5.43  (-2.99, 13.85) | 0.21 | 9.06  (3.26, 14.85) | 0.00* | 1.34  (-4.49, 7.17) | 0.65 | 3.23  (-3.90, 10.36) | 0.37 | 4.18  (-3.38, 11.74) | 0.28 |
| Bachelor’s degree | 18.01  (9.79, 26.23) | 0.00* | 15.74  (10.08, 21.39) | 0.00* | 6.60  (0.91, 12.28) | 0.02* | 6.21  (-0.75, 13.17) | 0.08 | 2.93  (-4.45, 10.31) | 0.44 |
| Master’s degree or above | 26.48  (17.64, 35.32) | 0.00* | 20.03  (13.95, 26.12) | 0.00* | 8.63  (2.51, 14.75） | 0.006* | 5.65  (-1.83, 13.14) | 0.14 | 2.24  (-5.70, 10.18) | 0.58 |
| **Maternal mood during gestation** |  |  |  |  |  |  |  |  |  |  |
| Anxiety score | -0.37  (-0.63, -0.11) | 0.005* | -0.21  (-0.39, -0.04) | 0.02* | -0.16  (-0.34, 0.009) | 0.06 | -0.44  (-0.65, -0.23) | 0.00* | -0.21  (-0.43, 0.01) | 0.06 |
| Depression score | -0.26  (-0.46, -0.06) | 0.01* | -0.19  (-0.33, -0.06) | 0.00* | -0.12  (-0.26, 0.01) | 0.07 | -0.42  (-0.58, -0.26) | 0.00* | -0.16  (-0.33, 0.01) | 0.07 |
| Pressure score | -0.65  (-0.92, -0.37) | 0.00* | -0.39  (-0.57, -0.20) | 0.00* | -0.13  (-0.49, -0.13) | 0.01* | -0.61  (-0.83, -0.40) | 0.00* | -0.06  (-0.29, 0.17) | 0.61 |

**P*<0.05. *CI* confidence interval, *BMI* body mass index, *SD* standard deviation, *FBG* fasting blood glucose.

Table 4: Associations of the characteristics of mother–infant pairs with WPPSI-IV scale scores at 4-5 years old.

| Characteristics | Verbal Comprehension Index | | Visual Space Index | | Fluid Reasoning Index | | Working Memory Index | | Processing Speed Index | | Full Scale IQ | |
| --- | --- | --- | --- | --- | --- | --- | --- | --- | --- | --- | --- | --- |
|  | *β* coefficient (95% CI) | *P* value | *β* coefficient (95% CI) | *P* value | *β* coefficient (95% CI) | *P* value | *β* coefficient (95% CI) | *P* value | *β* coefficient (95% CI) | *P* value | *β* coefficient (95% CI) | *P* value |
| **Exposure factor** |  |  |  |  |  |  |  |  |  |  |  |  |
| BMI (kg/m^2^) | -0.33  (-0.59, -0.06) | 0.02* | -0.33  (-0.60, -0.07) | 0.01* | -0.12  (-0.36, 0.11) | 0.31 | -0.11  (-0.35, 0.13) | 0.36 | -0.01  (-0.23, 0.21) | 0.91 | -0.26  (-0.49, -0.03) | 0.03* |
| Blood glucose (mmol/L) |  |  |  |  |  |  |  |  |  |  |  |  |
| FBG | -2.69  (-4.48, -0.91) | 0.003* | -3.20  (-4.98, -1.41) | 0.000* | -1.28  (-2.88, 0.31) | 0.11 | -1.23  (-2.84, 0.39) | 0.14 | -0.47  (-1.97, 1.03) | 0.54 | -2.21  (-3.78, -0.65) | 0.005* |
| 1 hour after a 75g  oral glucose load | 0.10  (-0.40, 0.61) | 0.69 | -0.59  (-1.09, -0.09) | 0.02* | -0.29  (-0.74, 0.16) | 0.21 | -0.47  (-0.92, -0.01) | 0.04* | -0.44  (-0.86, -0.02) | 0.04* | -0.40  (-0.83, 0.04) | 0.08 |
| 2 hours after a 75g  oral glucose load | -0.05  (-0.63, 0.55) | 0.88 | -0.38  (-0.97, 0.21) | 0.20 | -0.36  (-0.89, 0.16) | 0.17 | -0.69  (-1.22, -0.16) | 0.01* | -0.40  (-0.89, 0.09) | 0.11 | -0.41  (-0.93, 0.10) | 0.12 |
| Blood pressure (mmHg) |  |  |  |  |  |  |  |  |  |  |  |  |
| systolic pressure | -0.06  (-0.14, 0.02) | 0.16 | -0.08  (-0.16, -0.001) | 0.04* | -0.03  (-0.11, 0.03) | 0.29 | -0.06  (-0.13, 0.02) | 0.14 | 0.03  (-0.04, 0.10) | 0.39 | -0.05  (-0.12, 0.02) | 0.16 |
| diastolic pressure | -0.16  (-0.27, -0.04) | 0.008* | -0.18  (-0.30, -0.07) | 0.002* | -0.7  (-0.17, 0.04) | 0.22 | -0.06  (-0.17, 0.05) | 0.27 | -0.09  (-0.19, 0.01) | 0.07 | -0.15  (-0.25, -0.05) | 0.003* |
| PSQI total score | -0.14  (-0.48, 0.20) | 0.42 | -0.09  (-0.43, 0.25) | 0.61 | -0.40  (-0.71, -0.10) | 0.009* | -0.09  (-0.40, 0.22) | 0.57 | -0.10  (-0.38, 0.19) | 0.51 | -0.19(-0.49, 0.11) | 0.20 |
| smoke |  |  |  |  |  |  |  |  |  |  |  |  |
| No smoke | / | / | / | / | / | / | / | / | / | / | / | / |
| active smoking | 11.29  (-15.46, 38.05) | 0.41 | -13.48  (-40.24, 13.27) | 0.32 | 10.32  (-13.48, 34.13) | 0.40 | -11.04  (-35.12, 13.04) | 0.37 | -2.75  (-25.09, 19.58) | 0.81 | 2.63  (-20.72, 25.98) | 0.83 |
| **Basic information** |  |  |  |  |  |  |  |  |  |  |  |  |
| Maternal age (y) | 0.40  (0.17, 0.63) | 0.001* | 0.25  (0.02, 0.48) | 0.03* | -0.04  (-0.25, 0.16) | 0.67 | -0.05  (-0.26, 0.16) | 0.64 | -0.21  (-0.40, -0.01) | 0.04* | 0.13  (-0.08, 0.33) | 0.22 |
| Pre-pregnant BMI (kg/m^2^) | -0.17  (-0.45, 0.10) | 0.22 | -0.29  (-0.57, -0.02) | 0.04* | -0.09  (-0.33, 0.16) | 0.48 | -0.04  (-0.29, 0.20) | 0.73 | -0.00  (-0.23, 0.23) | 1.00 | -0.16  (-0.40, 0.08) | 0.19 |
| Gestational weight gain (g) | -0.000  (-0.002, 0.002) | 0.75 | 0.000  (-0.000, 0.004) | 0.10 | 0.000  (-0.000, 0.003) | 0.10 | 0.000  (-0.000, 0.003) | 0.08 | 0.000  (-0.001, 0.002) | 0.58 | 0.000  (-0.001, 0.003) | 0.17 |
| infant gender |  |  |  |  |  |  |  |  |  |  |  |  |
| male | / | / | / | / | / | / | / | / | / | / | / | / |
| female | 2.48  (0.80, 4.17) | 0.004* | -0.21  (-1.90, 1.48) | 0.81 | 1.25  (-0.25, 2.75) | 0.10 | 1.75  (0.23, 3.27) | 0.02* | 4.33  (2.94, 5.71) | 0.000* | 2.25  (0.78, 3.71) | 0.003* |
| Maternal education |  |  |  |  |  |  |  |  |  |  |  |  |
| Below high school degree |  |  |  |  |  |  |  |  |  |  |  |  |
| High school degree | 6.26  (0.56, 11.96) | 0.03* | 4.39  (-1.59, 10.36) | 0.15 | 4.49  (-0.80, 9.78) | 0.10 | 4.02  (-1.45, 9.48) | 0.15 | 5.47  (0.36, 10.58) | 0.04* | 6.01  (0.99, 11.03) | 0.02* |
| College degree | 10.86  (5.85, 15.87) | 0.000* | 5.48  (0.23, 10.73) | 0.04* | 5.07  (0.42, 9.72) | 0.03* | 3.35  (-1.45, 8.15) | 0.17 | 5.80  (1.31, 10.28) | 0.01* | 7.95  (3.54, 12.36) | 0.000* |
| Bachelor’s degree | 18.50  (13.61, 23.39) | 0.000* | 11.36  (6.23, 16.48) | 0.000* | 10.77  (6.23, 15.30) | 0.000* | 7.06  (2.37, 11.75) | 0.003* | 7.43  (3.05, 11.81) | 0.001* | 14.91  (10.60, 19.21) | 0.000* |
| Master’s degree or above | 22.03  (16.77, 27.29) | 0.000* | 12.98  (7.47, 18.50) | 0.000* | 12.35  (7.46, 17.23) | 0.000* | 9.19  (4.14, 14.24) | 0.000* | 7.59  (2.88, 12.31) | 0.002* | 17.67  (13.04, 22.30) | 0.000* |
| **Maternal mood during gestation** |  |  |  |  |  |  |  |  |  |  |  |  |
| Anxiety score | -0.20  (-0.35, -0.04) | 0.01* | -0.12  (-0.28, 0.03) | 0.12 | -0.26  (-0.39, -0.12) | 0.000* | -0.07  (-0.21, 0.07) | 0.33 | -0.07  (-0.20, 0.06) | 0.28 | -0.18  (-0.32, -0.05) | 0.01* |
| Depression score | -0.19  (-0.31, -0.07) | 0.002* | -0.11  (-0.23, 0.01) | 0.06 | -0.21  (-0.32, -0.11） | 0.000* | -0.08  (-0.18, 0.03) | 0.17 | -0.14  (-0.24, -0.04) | 0.01* | -0.19  (-0.29, -0.09) | 0.000* |
| Pressure score | -0.46  (-0.62, -0.30) | 0.000* | -0.14  (-0.31, 0.02) | 0.10 | -0.34  (-0.48, -0.19) | 0.000* | -0.17  (-0.32, -0.03) | 0.02* | -0.11  (-0.25, 0.03) | 0.11 | -0.36  (-0.50, -0.22) | 0.000* |

**P*<0.05. *CI* confidence interval, *BMI* body mass index, *SD* standard deviation, *FBG* fasting blood glucose.

Table 5: Multiple Linear Regression analysis of the association between maternal CVH levels during pregnancy and Bayley-III subscale scores at 2-3 years old (Unadjusted, n= 1007).

| Characteristics | Cognitive scale | | Language scale | | Motor scale | | Social–emotional scale | | Adaptive behavior scale | |
| --- | --- | --- | --- | --- | --- | --- | --- | --- | --- | --- |
|  | *β* coefficient (95% CI) | *P* value | *β* coefficient (95% CI) | *P* value | *β* coefficient (95% CI) | *P* value | *β* coefficient (95% CI) | *P* value | *β* coefficient (95% CI) | *P* value |
| BMI (kg/m^2^) | -0.32(-0.78, 0.14) | 0.178 | -0.20(-0.51, 0.11) | 0.210 | 0.04(-0.27, 0.35) | 0.808 | 0.10(-0.28, 0.47) | 0.606 | -0.13(-0.53, 0.26) | 0.500 |
| Blood glucose (mmol/L) |  |  |  |  |  |  |  |  |  |  |
| FBG | -7.62(-10.88, -4.36) | <0.001*^§^ | -3.65(-5.87, -1.43) | 0.001*^§^ | -1.75(-3.93, 0.43) | 0.115 | -1.16(-3.82, 1.49) | 0.390 | -1.19(-4.00, 1.61) | 0.404 |
| 1 hour after a 75g  oral glucose load | 2.11 (1.01, 3.20) | <0.001*^§^ | 1.43(0.68, 2.18) | <0.001*^§^ | 0.77(0.04, 1.51) | 0.040* | -0.64(-1.54, 0.25) | 0.160 | -0.37(-1.32, 0.58) | 0.442 |
| 2 hours after a 75g  oral glucose load | -0.75(-2.04, 0.54) | 0.255 | -0.98( -1.86, -0.10) | 0.029*^§^ | -0.47(-1.33, 0.39) | 0.286 | 0.54(-0.51, 1.59) | 0.311 | 0.59(-0.52, 1.70) | 0.299 |
| Blood pressure (mmHg) |  |  |  |  |  |  |  |  |  |  |
| systolic pressure | 0.24(0.08, 0.54) | 0.004*^§^ | 0.21(0.10, 0.32) | <0.001*^§^ | 0.20(0.09, 0.31) | <0.001*^§^ | 0.09(-0.04, 0.22) | 0.190 | -0.08(-0.22, 0.06) | 0.281 |
| diastolic pressure | -0.28(-0.51, -0.05) | 0.016*^§^ | -0.22(-0.37, -0.06) | 0.007*^§^ | -0.23(-0.39, -0.08) | 0.003*^§^ | -0.24(-0.43, -0.06) | 0.011* | -0.01(-0.21, 0.19) | 0.926 |
| PSQI total score | -1.07(-1.63, -0.50) | <0.001*^§^ | -0.83(-1.21, -0.44) | <0.001*^§^ | -0.56(-0.94, -0.19) | 0.003*^§^ | -0.19(-0.65, 0.27) | 0.418 | -0.42(-0.90, 0.07) | 0.092 |
| smoke |  |  |  |  |  |  |  |  |  |  |
| No smoke | / | / | / | / | / | / | / | / | / | / |
| active smoking | 38.50(-5.19, 82.19) | 0.084 | 6.58(-21.43, 38.14) | 0.582 | -6.31(-35.56, 22.94) | 0.672 | -10.29(-45.89, 25.31) | 0.571 | 17.45(-20.21, 55.10) | 0.363 |

**P*<0.05. ^§^ FDR< 0.05. *CI* confidence interval, *BMI* body mass index, *FBG* fasting blood glucose.

Table 6: Multiple Linear Regression analysis of the association between maternal CVH levels during pregnancy and WPPSI-IV subscale scores at 4-5 years old (Unadjusted, n= 1007).

| Characteristics | Verbal Comprehension Index | | Visual Space Index | | Fluid Reasoning Index | | Working Memory Index | | Processing Speed Index | | Full Scale IQ | |
| --- | --- | --- | --- | --- | --- | --- | --- | --- | --- | --- | --- | --- |
|  | *β* coefficient  (95% CI) | *P* value | *β* coefficient  (95% CI) | *P* value | *β* coefficient (95% CI) | *P* value | *β* coefficient (95% CI) | *P* value | *β* coefficient (95% CI) | *P* value | *β* coefficient (95% CI) | *P* value |
| BMI (kg/m^2^) | -0.21  (-0.50, 0.07) | 0.135 | -0.16(-0.44, 0.12) | 0.266 | -0.04(-0.29, 0.21) | 0.746 | -0.03(-0.29, 0.22) | 0.813 | 0.02(-0.22, 0.26) | 0.866 | -0.14(-0.38, 0.11) | 0.277 |
| Blood glucose (mmol/L) |  |  |  |  |  |  |  |  |  |  |  |  |
| FBG | -2.89(-4.88, -0.89) | 0.005*^§^ | -2.53(-4.52, -0.54) | 0.013* | -0.75(-2.53, 1.03) | 0.406 | -0.21(-2.02, 1.59) | 0.816 | 0.14(-1.53, 1.81) | 0.866 | -1.60(-3.34. 0.14) | 0.071 |
| 1 hour after a 75g  oral glucose load | 0.47(-0.20, 1.14) | 0.169 | -0.37(-1.04, 0.30) | 0.278 | -0.11(-0.71, 0.49) | 0.715 | -0.08(-0.69, 0.53) | 0.790 | -0.41(-0.97, 0.15) | 0.154 | -0.13(-0.72, 0.45) | 0.653 |
| 2 hours after a 75g  oral glucose load | 0.07(-0.72, 0.86) | 0.863 | 0.30(-0.49, 1.09) | 0.456 | -0.14(-0.84, 0.56) | 0.694 | -0.55(-1.26, 0.16) | 0.131 | -0.11(-0.77, 0.55) | 0.741 | -0.03(-0.72, 0.66) | 0.937 |
| Blood pressure (mmHg) |  |  |  |  |  |  |  |  |  |  |  |  |
| systolic pressure | 0.02(-0.08, 0.12) | 0.675 | 0.01(-0.09, 0.11) | 0.899 | -0.02(-0.11, 0.07) | 0.647 | -0.04(-0.13, 0.05) | 0.381 | 0.10(0.01, 0.18) | 0.022* | 0.03(-0.06, 0.11) | 0.552 |
| diastolic pressure | -0.15(-0.29, -0.01) | 0.042* | -0.14(-0.28, -0.001) | 0.052 | -0.03(-0.16, 0.09) | 0.593 | -0.02(-0.14, 0.11) | 0.789 | -0.15(-0.27, -0.04) | 0.011* | -0.14(-0.27, -0.02) | 0.022* |
| PSQI total score | -0.14(-0.48, 0.20) | 0.422 | -0.12(-0.46, 0.22) | 0.497 | -0.44(-0.75, -0.13) | 0.005*^§^ | -0.11(-0.44, 0.20) | 0.492 | -0.10(-0.39, 0.19) | 0.486 | -0.21(-0.52, 0.09) | 0.161 |
| smoke |  |  |  |  |  |  |  |  |  |  |  |  |
| No smoke | / | / | / | / | / | / | / | / | / | / | / | / |
| active smoking | 12.84(-13.88, 39.56) | 0.346 | -11.10(-37.81, 15.61) | 0.415 | 12.13(-11.75, 36.01) | 0.319 | -11.85(-36.05, 12.34) | 0.337 | -0.76(-23.15, 21.64) | 0.947 | 4.64(-18.71, 27.99) | 0.697 |

**P*<0.05. ^§^ FDR< 0.05. *CI* confidence interval, *BMI* body mass index, *FBG* fasting blood glucose.

Table 7: Multiple Linear Regression analysis of the association between maternal CVH levels during pregnancy and Bayley-III subscale scores at 2-3 years old (Adjusted, n= 1007).

| Characteristics | Cognitive scale | | Language scale | | Motor scale | | Social–emotional scale | | Adaptive behavior scale | |
| --- | --- | --- | --- | --- | --- | --- | --- | --- | --- | --- |
|  | *β* coefficient  (95% CI) | *P* value | *β* coefficient  (95% CI) | *P* value | *β* coefficient  (95% CI) | *P* value | *β* coefficient  (95% CI) | *P* value | *β* coefficient  (95% CI) | *P* value |
| BMI (kg/m^2^) | -0.76(-1.37, 0.36) | 0.09 | -0.27(-0.89, 0.35) | 0.39 | -0.20(-0.83, 0.42) | 0.52 | 0.36(-0.41, 1.12) | 0.36 | -0.26(-1.08, 0.55) | 0.53 |
| Blood glucose (mmol/L) |  |  |  |  |  |  |  |  |  |  |
| FBG | -5.66(-7.80, -1.75) | <0.00*^§^ | -2.23(-4.38, -0.08) | 0.04* | -1.04(-3.22, 1.14) | 0.35 | -0.65(-3.32, 2.02) | 0.63 | -1.28(-4.13, 1.56) | 0.38 |
| 1 hour after a 75g  oral glucose load | 1.16(0.16, 2.20) | 0.03* | 0.94(0.21, 1.67) | 0.01*^§^ | 0.37(-0.37, 1.11) | 0.32 | -0.60(-1.50, 0.30) | 0.19 | -0.11(-1.07, 0.85) | 0.82 |
| 2 hours after a 75g  oral glucose load | -0.98(-2.04, 0.32) | 0.11 | -1.11(-1.96, -0.27) | 0.01*^§^ | -0.52(-1.37, 0.34) | 0.24 | 0.27(-0.77, 1.32) | 0.61 | 0.59(-0.52, 1.70) | 0.30 |
| Blood pressure (mmHg) |  |  |  |  |  |  |  |  |  |  |
| systolic pressure | 0.21(0.07, 0.37) | <0.00*^§^ | 0.18(0.08, 0.29) | <0.00*^§^ | 0.18(0.08, 0.29) | <0.00*^§^ | 0.07(-0.06, 0.20) | 0.31 | -0.08(-0.25, 0.06) | 0.24 |
| diastolic pressure | -0.19(-0.39, 0.03) | 0.08 | -0.17(-0.32, -0.02) | 0.02*^§^ | -0.20(-0.35, -0.05) | 0.01*^§^ | -0.19(-0.18, 0.01) | 0.04* | -0.01(-0.21, 0.19) | 0.91 |
| Total score of PSQI | -0.95(-1.36, -0.26) | <0.00*^§^ | -0.81(-1.20, -0.42) | <0.00*^§^ | -0.55(-0.94, -0.15) | 0.01*^§^ | 0.29(-0.19, 0.78) | 0.23 | -0.20(-0.72, 0.31) | 0.44 |
| smoke |  |  |  |  |  |  |  |  |  |  |
| No smoke | / | / | / | / | / | / | / | / | / | / |
| active smoking | 29.18(-13.55, 66.00) | 0.16 | 1.72(-26.64, 30.08) | 0.91 | -9.90(-38.70, 18.90) | 0.50 | -4.12(-39.33, 31.09) | 0.82 | 23.05(-14.47, 60.58) | 0.23 |

**P*<0.05. ^§^ FDR< 0.05. *CI* confidence interval, *BMI* body mass index, *FBG* fasting blood glucose. Adjusted for confounders: infant’s sex, birth weight, maternal age, pre-pregnancy maternal BMI, maternal education level, and maternal mood during gestation including anxiety score, depression score, and pressure score.

Table 8: Multiple Linear Regression analysis of the association between maternal CVH levels during pregnancy and WPPSI-IV subscale scores at 4-5 years old (Adjusted, n= 1007).

| Characteristics | Verbal Comprehension Index | | Visual Space Index | | Fluid Reasoning Index | | Working Memory Index | | Processing Speed Index | | Full Scale IQ | |
| --- | --- | --- | --- | --- | --- | --- | --- | --- | --- | --- | --- | --- |
|  | *β* coefficient (95% CI) | *P* value | *β* coefficient (95% CI) | *P* value | *β* coefficient (95% CI) | *P* value | *β* coefficient (95% CI) | *P* value | *β* coefficient (95% CI) | *P* value | *β* coefficient (95% CI) | *P* value |
| BMI (kg/m^2^) | -0.30(-0.84, 0.24) | 0.28 | -0.31(-0.88, 0.26) | 0.28 | -0.13(-0.63, 0.37) | 0.62 | -0.41(-0.93, 0.11) | 0.12 | -0.12(-0.60, 0.37) | 0.63 | -0.35(-0.83, 0.12) | 0.15 |
| Blood glucose (mmol/L) |  |  |  |  |  |  |  |  |  |  |  |  |
| FBG | -1.30(-3.19, 0.60) | 0.18 | -1.58(-3.56, 0.39) | 0.12 | 0.33(-1.42, 2.08) | 0.71 | -0.35(-1.46, 2.17) | 0.70 | 0.54(-1.15, 2.23) | 0.53 | -0.27(-1.93, 1.39) | 0.75 |
| 1 hours after a 75g  oral glucose load | 0.08(-0.56, 0.72) | 0.81 | -0.67(-1.34, 0.003) | 0.05 | -0.31(-0.90, 0.29) | 0.31 | -0.28(-0.89, 0.33) | 0.37 | -0.44(-1.01, 0.13) | 0.13 | -0.45(-1.02, 0.11) | 0.11 |
| 2 hours after a 75g  oral glucose load | -0.01(-0.75, 0.73) | 0.98 | 0.25(-0.52, 1.02) | 0.53 | -0.15(-0.83, 0.54) | 0.68 | -0.52(-1.23, 0.19) | 0.15 | -0.14(-0.80, 0.52) | 0.67 | -0.06(-0.71, 0.59) | 0.86 |
| Blood pressure (mmHg) |  |  |  |  |  |  |  |  |  |  |  |  |
| systolic pressure | -0.01(-0.11, 0.08) | 0.78 | -0.00(-0.10, 0.09) | 0.94 | -0.04(-0.13, 0.05) | 0.36 | -0.05(-0.14, 0.04) | 0.24 | 0.09(0.005, 0.17) | 0.031* | 0.00(-0.08, 0.08) | 0.99 |
| diastolic pressure | -0.08(-0.21, 0.05) | 0.22 | -0.10(-0.23, 0.04) | 0.17 | -0.01(-0.11, 0.13) | 0.89 | 0.01(-0.11, 0.14) | 0.86 | -0.14(-0.26, -0.02) | 0.013* | -0.09(-0.20, 0.03) | 0.13 |
| PSQI total score | 0.07(-0.27, 0.42) | 0.68 | 0.003(-0.36, 0.36) | 0.99 | -0.19(-0.51, 0.12) | 0.23 | -0.02(-0.31, 0.35) | 0.89 | -0.01(-0.30, 0.32) | 0.95 | 0.01(-0.29, 0.31) | 0.95 |
| Smoke |  |  |  |  |  |  |  |  |  |  |  |  |
| No smoke | / | / | / | / | / | / | / | / | / | / | / | / |
| active smoking | 11.65(-12.29, 36.60) | 0.36 | -12.92(-39.00, 13.16) | 0.33 | 13.97(-9.10, 37.03) | 0.24 | -12.66(-34.75, 13.12) | 0.38 | 0.04(-22.26, 22.34) | 1.00 | 4.83(-17.06, 26.73) | 0.67 |

**P*<0.05. *CI* confidence interval, *BMI* body mass index, *FBG* fasting blood glucose. Adjusted for confounders: infant’s sex, birth weight, maternal age, pre-pregnancy maternal BMI, maternal education level, mode of delivery, and maternal mood during gestation including anxiety score, depression score, and pressure score.
